# A self-immolative Kdn-glycoside substrate enables high-throughput screening for inhibitors of Kdnases

**DOI:** 10.1093/glycob/cwae094

**Published:** 2024-11-21

**Authors:** Ali Nejatie, Cameron Proceviat, Christina Gros, Elizabeth Steves, Margo M Moore, David J Vocadlo, Andrew J Bennet

**Affiliations:** Department of Chemistry, Simon Fraser University, 8888 University Drive, Burnaby, BC V5A 1S6, Canada; Department of Chemistry, Simon Fraser University, 8888 University Drive, Burnaby, BC V5A 1S6, Canada; Department of Chemistry, Simon Fraser University, 8888 University Drive, Burnaby, BC V5A 1S6, Canada; Department of Biological Sciences, Simon Fraser University, 8888 University Drive, Burnaby, BC V5A 1S6, Canada; Department of Biological Sciences, Simon Fraser University, 8888 University Drive, Burnaby, BC V5A 1S6, Canada; Department of Chemistry, Simon Fraser University, 8888 University Drive, Burnaby, BC V5A 1S6, Canada; Department of Molecular Biology and Biochemistry, Simon Fraser University, 8888 University Drive, Burnaby, BC V5A 1S6, Canada; Department of Chemistry, Simon Fraser University, 8888 University Drive, Burnaby, BC V5A 1S6, Canada

**Keywords:** glycoside hydrolase, inhibitor, Kdnase, screen, self immolative

## Abstract

*Aspergillus fumigatus*, a filamentous fungus, is an opportunistic pathogen and the major causative agent of the often-fatal disease, invasive aspergillosis (IA). Current treatments for IA are limited due to their high toxicity and/or the emergence of drug resistance; therefore, a need exists for the development of new therapeutics to treat IA. The Kdnase produced by *A. fumigatus* plays a vital role in maintaining cell wall integrity. As there are no known Kdnases in humans, developing inhibitors of Kdnase from this fungal pathogen is a promising therapeutic approach. The rapid testing of enzymatic activity in a high-throughput screen of large chemical libraries can be an efficient way to find new small molecule lead compounds. Herein we show that a Kdn glycoside with a self-immolative cleavable aglycon is a practical and efficient substrate for a high throughput assay to identify Kdnase inhibitors. We optimized the activity assay and screened over 27,000 compounds from two bioactive chemical libraries as potential inhibitors, and we compared the hit compounds’ potency towards *Aspergillus terreus* and *Trichophyton rubrum* Kdnases, two other fungal Kdnases. We validated a number of hits and these small molecules are potential leads for the development of novel therapeutics to treat invasive aspergillosis.

## Introduction

*Aspergillus fumigatus* (*Af*) is the most common causative agent of the fungal infection invasive aspergillosis (IA), which frequently affects immunocompromised individuals (Latge 1999; Dagenais and Keller 2009). Mortality rates range from 30%–90% with *Af* causing more than 60% of infections in immunocompromised patients (Rybak et al. 2019), and recently, *Af* was identified as the causative agent in critically ill COVID-19 patients suffering from IA (Benedetti et al. 2022). A limited number of treatments for IA are available likely because *A. fumigatus* was, until the late 20^th^ century, considered a weak pathogen (Latge 1999). This view slowed development of new treatments against *Af*. Moreover, existing therapies have significant adverse effects arising either from their toxicity or the emergence of drug resistance. For example, liposomally formulated Amphotericin B is the standard of care, however, at therapeutic doses it is associated with nephrotoxicity (Latge 1999). Triazole-based drugs are also used to treat IA, however, strains of *Af* resistant to these therapeutics continue to emerge limiting the effectiveness of these drugs (Rybak et al. 2019). As a result, new therapeutics are needed to treat IA. The Kdnase produced by *A. fumigatus* (*Af*K) (Telford et al. 2011; Yeung et al. 2013; Nesbitt et al. 2018) is involved in the maintenance of structure and function of the fungal cell wall, however, its natural substrate remains unknown (Nesbitt et al. 2018). Immunocompromised mice that were infected with wild-type (WT) *A. fumigatus* spores (Nesbitt et al. 2018) followed by treatment with Amphotericin B showed complete mortality, whereas, those infected with *Af*K KO spores, and then treated with Amphotericin B, showed an improved survival rate (Nesbitt et al. 2018). Taken together, these results suggest that inhibitors of *Af*K might have potential as sensitizers that could help combat *A. fumigatus*. There are, however, no effective inhibitors of *Af*K, which motivated us to pursue development of a high-throughput screen (HTS) to discover new Kdnase inhibitors that might be exploited in this regard. While several screening approaches have been pursued to identify inhibitors of *A. fumigatus* growth, such as the resazurin method that simply monitors cell viability (Monteiro et al. 2012; Smith et al. 2016). Interestingly, using this assay Lockhart et al discovered new classes of inhibitors of chitinase A1 (*Af*ChiA1), which is involved in the structure and function of the fungal biofilm in *A. fumigatus* (Lockhart et al. 2014). This observation suggests that the carbohydrate processing enzymes of *A. fumigatus* represent a promising set of targets. *Af*K, in particular, has an activity that is not found within humans, making this a target that could deliver selective antagonists without adverse effects to patients. Herein, we report the synthesis of an *O*-alkyl Kdn glycoside that contains a self-immolative aglycon, which following glycosidic bond cleavage can undergo a non-enzymatic cyclization to generate a fluorescence signal. Following assay optimization we used this substrate in two high-throughput screens to locate Kdnase inhibitors.

## Results and discussion

**Fig. 1:**
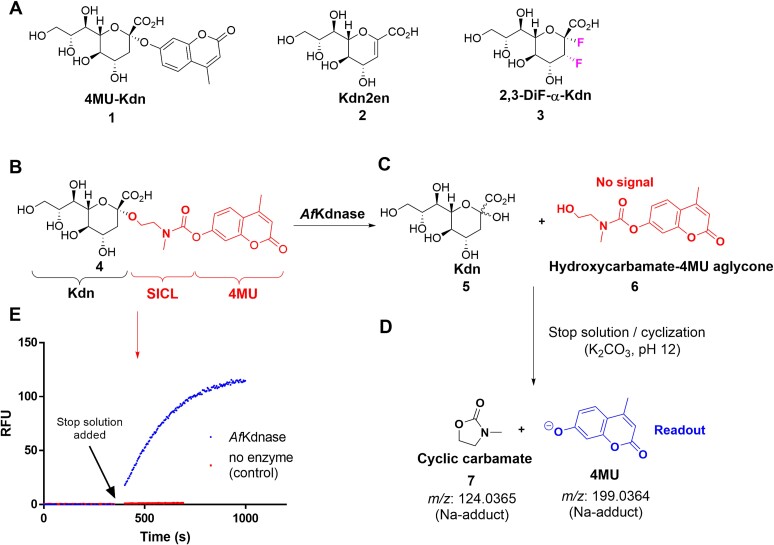
**Structures of Kdn-based probes and inhibitors, and the mechanism of action for HTS substrate 4**. A) Structure of 4MU-Kdn **1**, competitive inhibitor Kdn2en **2**, and irreversible inactivator 2,3-diF-α-Kdn **3**; B) structure of self-immolative probe **4**; C) *Af*K-catalyzed hydrolysis of **4** to give Kdn **5** and intermediate **6**; D) base promoted cyclization of **6** to give cyclic carbamate **7** and the strongly fluorescent 4MU anion; and E) plot of fluorescent signal production in the presence of enzyme (upper curve), following a pH jump, and the negative control showing a lack of signal in the absence of added *Af*K (flat line).

When developing a HTS assay of enzymatic activity, an optimal substrate should possess an intrinsically low background signal to obtain good assay statistics and avoid false positive hits (Inglese et al. 2007). We avoided using aryloxy Kdn glycosides due to their known high intrinsic reactivity (Nejatie et al. 2022), which is partially caused by the lack of an electron withdrawing C3 substituent adjacent to the anomeric center (Ashwell et al. 1992; Dookhun and Bennet 2005; Nejatie et al. 2022). Specifically, the substrate 4-methylumbelliferyl 3-deoxy-d*-glycero*-d-*galacto*-non-2-ulopyranosidonic acid (4MU-Kdn, **1**, Fig. 1A) exhibits a significant non-enzymatic background signal during the total incubation time required for the HTS. Our first attempt to circumvent the issue of non-enzymatic hydrolysis involved synthesizing aryl Kdn thioglycosides, which are much less hydrolytically labile (Narine et al. 2006; Nejatie et al. 2021a). Unfortunately, *Af*K was insufficiently active towards hydrolysis of aryl Kdn thioglycosides for these compounds to be useful in a HTS (Nejatie et al. 2021a). Therefore, we turned our attention to the use of a self-immolative substrate (Kdn-SICL-4MU, **4**, Fig. 1B) that following glycosidic bond cleavage can undergo a non-enzymatic cyclization to liberate a cyclic carbamate **7**, and a fluorophore, 4MU, (Fig. 1D) (Alouane et al. 2015; Nasseri et al. 2018). We opted for an *O*-alkyl Kdn glycoside, which we judged would be kinetically stable in buffered solutions and therefore produce an insignificant non-enzymatic background signal, for the self-immolative cleavable linker (SICL) (Fig. 1E). Here we detail the synthesis of an alkylamino carbamate-based substrate (**4**) that is converted by enzyme-catalyzed cleavage to a product that readily undergoes base promoted cyclization to release a fluorescent reporter (Shan et al. 1997; Alouane et al. 2015).

We selected 4-methylumbelliferone (4MU) as the fluorescence reporter due to its well established physicochemical and photophysical properties (Fig. 1D).

**Scheme 1:**
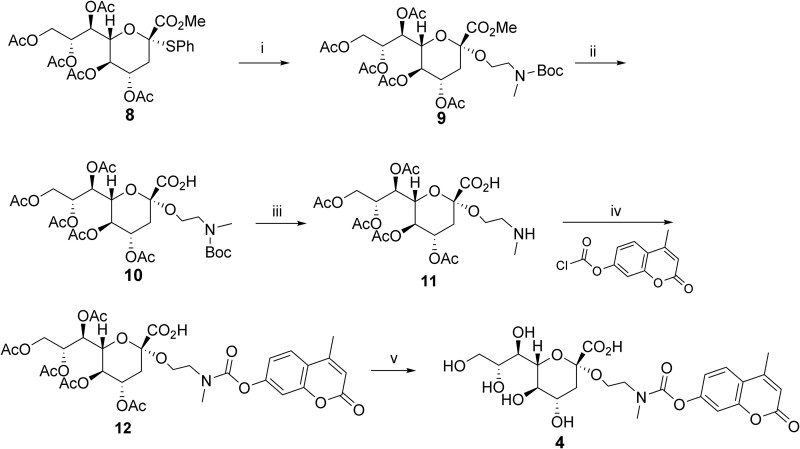
**Synthetic route for the production of Kdn glycoside 4**. Reagents and conditions: (i) *N*-Boc-*N*-methyl-2-aminoethanol, IBr, AgOTf in CH_2_Cl_2_/CH_3_CN (2:1 v/v) rt, 84%, α:β 9:1; (ii) LiI/pyridine, 120 °C, 20 h, 56%; (iii) TFA in CH_2_Cl_2_, rt, 2 h. (iv) 4-methylumbelliferyl chloroformate, DIPEA in CH_2_Cl_2_, rt 44 h, 78% over two steps. (v) K_2_CO_3_ in CH_3_OH, rt, 8 h, 76%. Boc = *^t^*butoxycarbonyl, DIPEA = diisopropylethylamine, TFA = trifluoroacetic acid.

For the self-immolative linker we opted to use a carbamate group as they are generally inert to attack by hydroxyl nucleophiles in buffered aqueous solutions. In addition, we incorporated a two carbon alkyl linker into our design as five-membered ring cyclizations are generally facile. Therefore, we coupled commercially available *N*-methyl-*N*-Boc-2-aminoethanol and thioaryl Kdn donor **8** (Terada et al. 1993) using known glycosylation conditions (Scheme 1) (Caraballo et al. 2015) to afford glycoside **9** with excellent stereoselectivity and yield. Of note, a secondary amine is needed for formation of the necessary carbamate linker to avoid an E1_CB_ elimination reaction occurring during subsequent deprotection. (Scheme S1 and S2, Supporting Information). Initial efforts at deprotection of the amine, prior to installation of the aryl carbamate, gave lactam **19** (Scheme S3, Supporting Information) via an intramolecular cyclization of the secondary amine onto the anomeric ester.

To circumvent this lactamization we hydrolyzed the methyl ester of **9** using lithium iodide in pyridine (Bartlett and Johnson 1970) to obtain acid **10**. After separating the undesired β-glycoside, we only now removed the *N*-Boc group under acidic conditions to give secondary amine **11**, which was immediately coupled to 4-methylumbelliferyl chloroformate (Prost et al. 2014). This reaction sequence gave protected glycoside **12** in a good yield. Finally, deacetylation of **12** under mildly basic conditions furnished Kdn glycoside **4** (Scheme 1).

With Kdn glycoside **4** in hand, we began assay development. Our initial tests, using ^1^H NMR and mass spectroscopy, showed that *Af*K-catalyzed cleavage of **4** could be driven to completion under the mildly acidic conditions that favour enzyme activity (Fig. S1, Supporting Information). Of note, the liberated aglycon **6** did not cyclize under these conditions (Fig. 1C and E). We reasoned that hydroxyl group activation is required for cyclization. We therefore allowed the enzymatic reaction to proceed for 5 min and then raised the pH of the solution to a pH of ≈ 10 by addition of a solution of K_2_CO_3_ (Fig. 1D). We opted for this raised pH value because it would both facilitate cyclization releasing the 4MU reporter and also terminate the enzyme-catalyzed substrate hydrolysis. Using this approach, we observed a significant increase in fluorescence attributable to *Af*K-catalyzed hydrolysis of substrate **4**. Also, the no enzyme control displayed no discernable increase in fluorescence following addition of the basic stop solution (Fig. 1E). That is, in the absence of Kdnase, no hydroxycarbamate aglycon **6** is formed. Based on these data we concluded that, under our assay conditions, probe **4** is enzymatically cleaved and that, following a pH jump to a basic value, cyclization occurs to liberate the 4MU fluorophore. Notably, addition of the carbonate stop solution results in a first-order cyclization of the liberated aglycon, a process that occurs with a half-life (*t*_1/2_) of approximately 2.5 min. Thus, >98% of the fluorescence signal is produced within 15 min following addition of the basic buffer (Fig. S2, supporting information). Beneficially, the jump in pH upon addition of basic carbonate buffer ensures that the 4MU fluorophore (p*K*_a_ = 8.4) is predominantly in its intensely fluorescent phenoxide form, which thereby enhances the assay sensitivity.

Next, we measured the Michaelis–Menten parameters for *Af*K-catalyzed hydrolysis of **4** so as to guide assay optimization for the downstream HTS. Specifically, we added stop solution to the *Af*K-catalyzed reaction at various times and then, after cyclization we measured the fluorescence, which we converted to product concentration using a standard calibration curve (Figs S3–S5, Supporting Information). As expected, our alkyl Kdn glycoside undergoes enzyme-catalyzed hydrolysis with lower rate constants than those for the activated Kdn glycoside 4MU-Kdn. Specifically, the second-order rate constant (*k*_cat_/*K*_m_) for the *Af*K-catalyzed hydrolysis of **4** is (4.0 ± 0.8) × 10^3^ M^−1^ s^−1^, while that for 4MU-Kdn (**1**) is (1.8 ± 0.1) × 10^5^ M^−1^ s^−1^ (Yeung et al. 2013) (Table S1, Supporting Information).

**Fig. 2:**
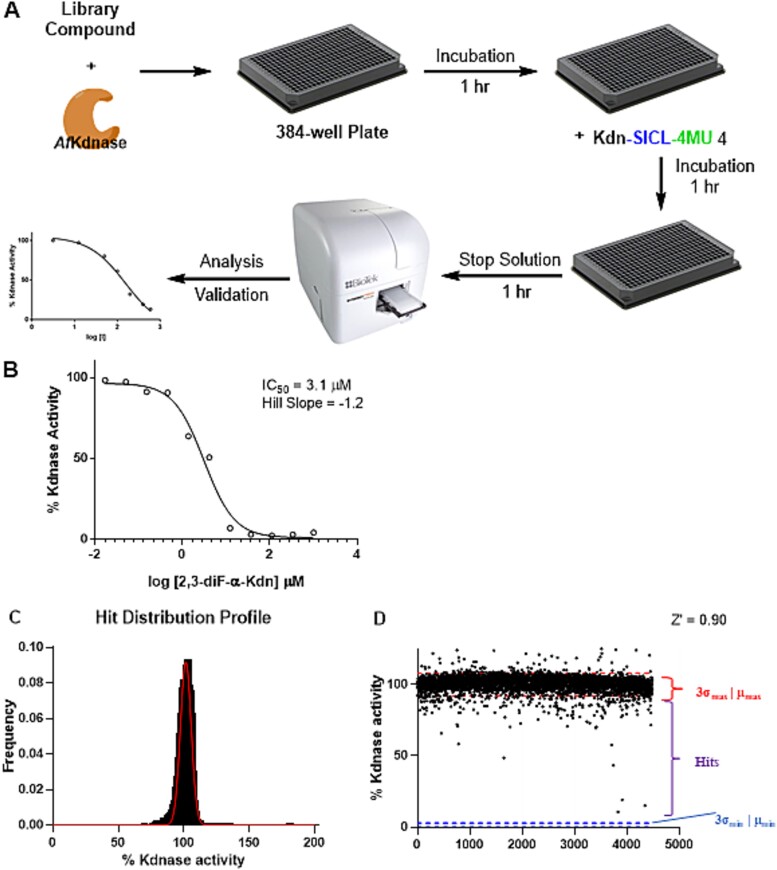
**Overview of the HTS assay of the TargetMol library**. A) Schematic overview of fluorescent-based high-throughput screen of *Af*K activity using Kdn glycoside **4**, B) evaluation of apparent IC_50_ value following incubation (10 min) of the known inactivator 2,3-diF-α-Kdn **3**, C) hit distribution profile of the TargetMol library against *Af*K, and D) activity for 4,480 bioactive compounds, μmax represents the mean signal of uninhibited positive control and μmin represents the mean signal of the negative control in the absence of enzyme.

We next miniaturized our assay for use in a 384-well microplate format by optimizing the quantity of detergent and checking for fluorescent signal stability (Figs 2A,S6 and S7 Supporting Information). In each well, we added a solution of buffer containing *Af*K (50 nM) and then added a library compound using a pin tool. We included both detergent (0.2% (v/v) Tween-20) and BSA (0.01% BSA) to reduce false positives arising from aggregation prone compounds (Feng et al. 2007). We then equilibrated the mixture prior to the addition of substrate **4**, and the resultant solution was incubated for 1 h after which stop solution was added. To ensure cyclization was complete, the mixture was allowed to react for 1 h prior to measuring the fluorescence. Candidate hits identified in this primary screen were cherry picked and delivered into a gold plate that was used to confirm hits.

As a positive control within the screen, we used the known irreversible inhibitor 2,3-diF-Kdn **3** (Fig. 1A) (Watts et al. 2006), which inactivates *Af*K by forming a covalent adduct with the active site nucleophile (Watts et al. 2006; Telford et al. 2011; Khazaei et al. 2015). That is, we found that a 2,3-diF-Kdn (**3**) concentration of 30 μM when reacted with *Af*K for 10 min resulted in a fluorescent signal equal to <10% that of the uninhibited sample.

**Fig. 3:**
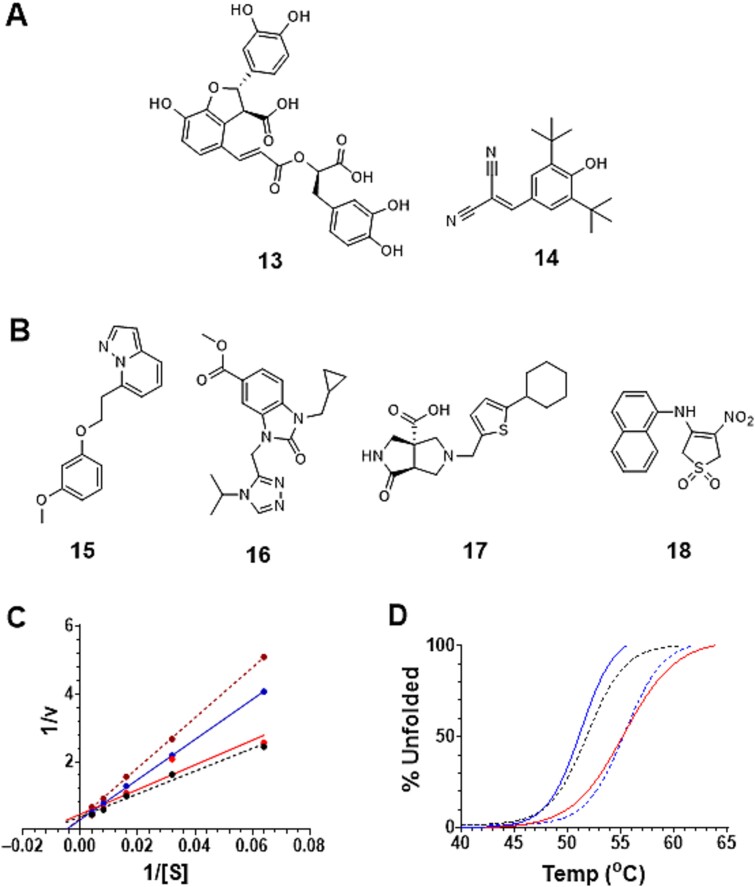
**Structures and inhibition plots of identified hits**. A) Structures of the two identified hits from the TargetMol library; lithospermic acid **13** and Tyrphostin A9 **14**, B) structures of positive hits from the Chembridge Corp library **15**–**18**, C) Lineweaver-Burk plot for the inhibition of *Af*K by **17** (upper dashed line 15.6 μM, upper solid line 7.8 μM, lower solid line 1.95 μM, and black dashed line no inhibitor), and D) differential scanning fluorometry-based assay for inhibitors (**13**, solid blue line), **17** (lower dashed line), Kdn2en **2** (solid red line), and control (DMSO dashed black line) on the thermal stability of folded *Af*K.

Using these assay conditions, we performed a pilot screen on a set of 4,480 known bioactive compounds. We obtained an overall Z' score of 0.90 over the entire assay with a normal hit distribution profile. Given the large separation band, we set the cut-off for hits to be 60% residual Kdnase activity, which yielded an overall fraction of hits of 0.15% (Fig. 2C and D). These seven compounds were then subjected to a repeat assay to verify their inhibitory activity. Of these hits we confirmed two that inhibit *Af*K; lithospermic acid **13**, a natural product extract from *Radix salvia Miltiorrhizae,* which has therapeutic effects towards angiocardiopathy (Wang et al. 2015) and Tyrphostin A9 **14**, a known protein tyrosine kinase inhibitor (Fig. 3A) (Gazit et al. 1989). The remaining five original hits did not demonstrate inhibition in the repeat assay.

We purchase fresh commercial material for compounds **13** and **14** and performed inhibition assays using recombinant *Af*K (Methods, Fig. S8, Supporting Information). We determined IC_50_ values in quadruplicate using 4MU-Kdn as the substrate (Yeung et al. 2013). By inspecting the Lineweaver-Burk plots for inhibition by **13** and **14**, we established these compounds are competitive inhibitors (Fig. S8, Supporting Information). Of note, lithospermic acid (**13**), the most potent hit, displayed a 24-fold lower IC_50_ value than that reported for the Kdn glycal (Kdn2en **2**, Table S2, Supporting Information) (Yeung et al. 2013). We submitted both compounds to a pan-assay interference compounds (PAINS) filter, a compilation of chemical substructures known to show promiscuous biological activities (Baell and Nissink 2018). Compound **14** was flagged as likely having PAIN activity and thus, we stopped investigating this material. We next moved to examine whether **13** exhibited time-dependant inhibition, which is often a sign of inhibition arising in a non-specific manner, and gratifyingly we found no time dependent changes in calculated IC_50_ values (Fig. S9, Supporting Information). These observations support our conclusion that **13** is a competitive inhibitor of *Af*K. Interestingly, inhibitor **13** contains two carboxylic acid groups, a functionality that features prominently in the natural substrate Kdn and which is engaged in numerous interactions within the active site including with an arginine triad that is important for substrate binding (Lentz et al. 1987; Telford et al. 2011). Ideally, we would have performed a structure–activity relationship (SAR) study on this validated hit to identify the chemical groups responsible for providing the critical interactions that give rise to inhibition. However, lithospermic acid (**13**) is a trimeric condensation product of caffeic acid and is difficult to modify for SAR studies. We found that caffeic acid itself, however, inhibits *Af*K, albeit only weakly with an IC_50_ value of ≈ 0.5 mM (Fig. S10, Supporting Information). Recently, we reported that the pathogenic fungi *Aspergillus terreus* and *Trichophyton rubrum* produce Kdnases (*At*K and *Tr*K), (Nejatie et al. 2021b) and as both of these enzymes belong to the same glycoside hydrolase family (GH33) as *Af*K, we decided to test if these enzymes, which possess structurally similar active sites, are also inhibited by **13**. We performed standard inhibition assays as described earlier using inhibitor **13** and these recombinant enzymes. Strikingly, we found that both *At*K and *Tr*K were competitively inhibited by **13** with similar IC_50_ values to those measured for *Af*K (Table 1, Fig. S11, Supporting Information). We also tested inhibitor **13** with the sialidase *Mv*NA, the closest structural homologue to *Af*K, that is known to cleave a multitude of substrates including Kdn glycosides (Telford et al. 2011). We note that although **13** inhibits *Mv*NA, it does so with a 30-fold increase in IC_50_ value, indicating that this inhibitor is more selective towards Kdnase processing enzymes.

**Table 1:** Kinetic parameters for sialidase inhibition. Listed are the IC_50_, K*_i_* and hill coefficient for the inhibitors lithospermic acid (**13**) and **17** against the Kdnases from *Af*, *At*, and *Tr*, and the neuraminidase *Mv*NA. Assays were performed at 37 °C using Kdn4MU as substrate in a NaOAc buffer (100 mM, pH 4.0) for the Kdnases and at pH 5.5 for *Mv*NA, all buffers contained 0.01% BSA. All data were fit to a standard IC_50_ equation using the computer program prism 7.0, while *K*_i_ values were calculated using equation *K*_i_ = IC_50_/(([S]/*K*_m_) + 1).

**Kdnase source**	**Inhibitor**	**IC**_**50**_ **(μM)**	***K***_**i**_ **(μM)**	**Hill**
*A. fumigatus*	**13**	22.8 ± 1.0	18.7	−1.32
	**17**	115 ± 2	74.7	−0.81
*A. terreus*	**13**	14.4 ± 0.07	11.1	−1.36
	**17**	50.0 ± 1.2	38.5	−0.88
*T. rubrum*	**13**	11.8 ± 0.1	9.2	−0.80
	**17**	93.3 ± 0.8	73.3	−1.11
*Micromonospora viridifaciens*	**13**	347 ± 2	224	−0.90
	**17**	6.6 ± 0.6	4.6	−0.89

After completing the pilot screen, we conducted a second HTS on a library of 23,040 structurally diverse compounds. In this screen, we again obtained a favorable overall Z' score of 0.87 with a clear hit distribution profile (Fig. S12, Supporting Information). With a similar large separation band and tight controls as the pilot screen, we decided to investigate hits below an 80% cutoff for residual Kdnase activity, which translated to an overall percentage of hits to be approximately 0.01%. The four compounds selected in this way were subject to analysis using the PAINS filter and all four showed no liabilities (Fig. 3B) (Baell and Nissink 2018). We purchased compounds **15**–**18** and performed standard inhibition assays using recombinant *Af*K. We determined IC_50_ values using 4MU-Kdn **1** as substrate and established that only compound **17** inhibited *Af*K and moreover it is a competitive inhibitor (Fig. 3C). We note that the computed IC_50_ value is 5-fold lower than that reported for the Kdn glycal Kdn2en **2** (Yeung et al. 2013). We then repeated these activity assays for **17** against *Tr*K, *At*K and *Mv*NA. Notably, all three compounds; **2**, **13**, and **17** posses at least one carboxylic acid that, when ionized, can engage the catalytically important and highly conserved arginine triad in the active site of *Af*K (Telford et al. 2011; Baell and Nissink 2018). This situation is clearly repeated in all four GH33 enzymes (*Af*K, *At*K, *Tr*K, and *Mv*NA) (Newstead et al. 2005; Nejatie et al. 2021a), where these carboxylic acid-based compounds exhibited inhibitory effects at low μM concentrations (Tables 1 and Fig. S13, Supporting Information).

We next used a separate biophysical technique, differential scanning fluorimetry (DSF), to confirm that these compound bind to the various Kdnases. Specifically, we measured protein melting temperatures in the presence of Kdn2en (**2**) and candidate ligands **13** and **17** (Fig. 3D). We note that **2** and inhibitor **17** both show positive unfolding temperature shifts, whereas, compound **13** had no effect on the stability of *Af*K. We repeated this DSF assay on *At*K and *Tr*K and we observed similar trends in thermal shifts (Fig. S14 and Tables S2,S3 and S4, Supporting Information). The absence of apparent stabilization of *Af*K as seen in these DSF experiments suggests this compound may not bind as a reversible inhibitor to the enzyme. Indeed, while lithospermic acid (**13**) is not formally classified as a PAINS compound, it does contain two catechol units, which have been shown to sometimes exert non-specific effects leading to inhibition in the low micromolar range (Baell 2016). While the presence of two carboxylates within this molecule, coupled with the apparent competitive mode of inhibition (Fig. S8, Supporting Information) and the absence of time-dependent inhibition of *Af*K (Fig. S9, Supporting Information), suggest that binding to the active site occurs in a reversible manner, we cannot definitively rule out an alternative non-specific mode of inhibition. Accordingly, further follow up on this compound would need to be pursued with an awareness of this possibility.

In summary, the two confirmed positive HTS hits are potential lead compounds, albeit with the reservation noted above for lithospermic acid (**13**). Ultimately, these compounds provide us with insights into the types of chemical structures that can be used for the development of more potent Kdnase inhibitors. In addition, the results from these screens illustrate Kdn glycoside **4** can be used in a robust assay that could be applied to screen additional libraries in order to identify new inhibitors of *Af*K, other Kdnases or, indeed, sialidases.

## Materials and methods

All buffers were purchased from Sigma and used without further purification. Milli-Q water (18.2 MΩ cm) was used for kinetic and product study experiments. Potassium Carbonate stop solution was made to a 200 mM or 1 M solution. All other salts used in the hydrolysis runs were of analytical grade and were used without purification. ^1^H NMR (400 MHz) and ^13^C NMR (101 MHz) spectra were recorded in CDCl_3_ (calibrated at δ_C_ 77.0 ppm, and using residual CHCl_3_ at δ_H_ 7.26 ppm), CD_3_OD (calibrated at δ_C_ 49.0 ppm, and using residual CH_3_OD at δ_H_ 3.31 ppm). All shifts are reported in parts per million (ppm). All coupling constants (*J*) are reported in hertz (Hz) and were obtained from analysis of ^1^H NMR spectra. Multiplicities are abbreviated as singlet (s), broad singlet (bs), doublet (d), doublet of doublet (dd), triplet (t), doublet of triplet (dt), and multiplet (m). Anhydrous solvents were purchased commercially from Sigma Aldrich. Organic solutions were dried over Na_2_SO_4_ and concentrated under reduced pressure. All products were dried under high vacuum. Thin layer chromatography (TLC) was performed on aluminum plates coated with silica gel and charred with Seebach stain (Seebach et al. 1987). Compounds were purified by Combiflash Rf unless otherwise stated. Compounds **3** (Khazaei et al. 2015) and **8** (Terada et al. 1993) were prepared from known literature procedures. *Af*K was expressed and purified as described by Telford et al. (Telford et al. 2011). NMR spectra of new compounds are given in Supplementary Materials Figures S15-S22.

### Kdnase activity assays

The activity of *Af*K was determined by measuring cleavage of the synthetic Kdnase substrate Kdn-SICL-4MU **4** (Fig. 1). Substrate **4** was incubated at rt for 10 min followed by the addition purified recombinant enzyme. A stop solution containing 100 mM K_2_CO_3_ pH ≈ 12 was added at a series of time points starting from 5, 10, 15, 30, 45, 60, 120, 180 min. The mixtures were allowed to react an additional 30 mins to ensure cyclization was completed before taking measurements. The substrate concentration used ranged of 2–2,000 μM and the amount of 4-methylumbelliferone (4MU) released from **4** was determined using a fluorescence spectrophotometer (BioTek Neo 2) at excitation and emission wavelengths of 360 and 450 nm, respectively. The control reactions were set up in cuvettes by adding 100 μM Kdn-SICL-4MU, and the specific reaction buffer for a total volume of 200 μL without enzyme.

### HTS assay

To each well in a 384-well microplate, enzyme (20 μL of a 50 nM stock solution) in reaction buffer (100 mM NaOAc, 0.2% Tween-20, 0.01% BSA, pH 4) was added to a library compound (200 nL of 20 μM TargetMol, 10 μM Chembridge), and this mixture was incubated at rt for 1 h. Then, substrate **4** (20 μL of a 50 μM stock) was added and the resultant mixture was incubated at rt for an additional 1 hr. Following addition of stop solution (40 μL of 1 M aqueous K_2_CO_3_, pH ≈ 12) to the mixture the plate was incubated for 1 h at rt. Next, the fluorescence for each well was measured (BioTek Neo 2) using an excitation and an emission wavelength of 360 and 450 nm, respectively. The collected data was analyzed using Prism 7.0. We used the known mechanism-based inactivator 2,3-diF-Kdn (**3**) as the positive control (Watts et al. 2006; Khazaei et al. 2015). That is, using 2,3-diFKdn at a concentration of 30 μM, following a 10 min incubation, gives a background signal that equates to >90% inhibition.

### Inhibition assay

Commercially purchased inhibitors (**15**–**18**) were tested for inhibition of *Af*K using Kdn-4MU (2–2,000 μM), and the concentration range of inhibitor used were from 3.9–250 μM. Each 200 μM reaction mixture was incubated at rt for 2 min prior to the addition of purified sialidase. Inhibitor was added to the reaction mixture and allowed to incubate at rt between 0–60 min in 100 mM sodium acetate buffer pH 4.0 and 0.01% BSA prior to the addition of substrate. The kinetic parameters were determined from initial rate measurements using a substrate concentration of *K*_m_/4. The progress of the reaction was continuously monitored for 15 min using a fluorescence spectrophotometer (BioTek Neo 2) and excitation and emission wavelengths of 360 and 450 nm, respectively. The collected data were fitted to a standard binding equation (Prism 7.0) to give IC_50_ values, and *K*_i_ values were calculated using the equation *K*_i_ = IC_50_/(([S]/*K*_m_) + 1) (Segel 1975).

### Methyl (*N*-methyl-*N*-Boc-2-aminoethyl 4,5,7,8,9-penta-*O*-acetyl-3-deoxy-d-*glycero*-α-d-*galacto*-non-2-ulopyranosid)onate(9)

Kdn donor **8** (Terada et al. 1993) (5.0 g, 8.35 mmol), *N*-Boc-*N*-methyl-2-aminoethanol (2.2 g, 12.52 mmol, 1.5 equiv.), and activated 4 Å molecular sieves (11 g) were dissolved in a mixture of dry CH_3_CN/CH_2_Cl_2_ (165 mL/85 mL) and the mixture was stirred at rt under N_2_ for 30 mins. The temperature of the mixture was lowered to −78 °C and it was protected from light. Then a solution of AgOTf (4.3 g, 16.7 mmol, 2 equiv.) in dry CH_3_CN (10 mL) was added followed by a 1 M solution of IBr in CH_2_Cl_2_ (12.5 mL, 1.5 equiv.). The resulting mixture was stirred at −78 °C for 4 h when it was quenched by the addition of DIPEA (8.7 mL, 50.1 mmol, 6 equiv.). This mixture was filtered over a pad of Celite^®^, which was subsequently washed with CH_2_Cl_2_ (200 mL). The combined filtrate and washing were extracted with saturated solutions of Na_2_S_2_O_3_ (100 mL) and NaHCO_3_ (2 × 100 mL). The combined organic layers were dried (Na_2_SO_4_) and concentrated to give a residue that was purified by column chromatography (EtOAc/Hexanes 4:6 → 1:1 v/v) to give **9** (4.4 g, 81%, 9:1, α:β) as a pale yellow amorphous solid. (~1:2 mixture of rotamers) ^1^H NMR (400 MHz, CDCl_3_) δ_H_ 5.39 (ddd, *J* = 9.2, 4.8, 2.6 Hz, 1H, H-8), 5.32 (dd, *J* = 9.2, 2.1 Hz, 1H, H-7), 4.95–4.75 (m, 2H, H4, H-5), 4.24 (dd, *J* = 12.5, 2.4 Hz, 1H, H-9a), 4.17–4.08 (m, 2H, H-6, H-9b), 3.85–3.79 (m, 1H, -OCH*H*CH_2_NCH_3_COO-), 3.78 (s, 3H, C=OC*H*_3_), 3.73 (t, *J* = 5.3 Hz, 1H, OCH_2_CH*H*NCH_3_COO-), 3.46–3.35 (m, 2H, -OC*H*_2_CH_2_NCH_3_COO-), 3.33–3.24 (m, 1H, -OCH_2_C*H*HNCH_3_COO-), 2.87 (s, 3H, -NC*H_3_*), 2.65 (dd, *J*_3eq,3ax_ = 13.0, *J*_3eq,4_ = 4.3 Hz, 1H, H-3_eq_), 2.14 (s, 3H, C=OC*H*_3_), 2.09 (s, 3H, C=OC*H*_3_), 2.02 (s, 3H, C=OC*H*_3_), 1.99 (s, 3H, C=OC*H*_3_), 1.98 (s, 3H, C=OC*H*_3_), 1.88 (t, *J*_3eq,3ax_ = 12.3 Hz, 1H, H-3_ax_), 1.44 (s, 9H, -C(C*H*_3_)_3_). ^13^C NMR (101 MHz, CDCl_3_) δ_C_ 170.54 (C=O), 170.02 (C-1), 169.80 (C=O × 2), 169.74 (C=O), 167.81 (C=O), 98.67 (C-2), 79.32 (4 °C), 71.20 (C-6), 69.29 (C-4), 67.87 (C-5), 66.56 (C-7), 64.11 (-O*C*H_2_CH_2_NCH_3_COO-), 62.09 (C-9), 52.75 (C=O*C*H_3_), 51.51 (-OCH_2_*C*H_2_NCH_3_COO-), 37.61 (C-3), 35.46 (-N*C*H_3_), 28.39 (-C(*C*H_3_)_3_), 21.02, 20.76, 20.64, 20.61, 20.57(-C=O*C*H_3_); HRMS-ESI (m/z) Calcd for C_28_H_43_NO_16_ ([M + Na])^+^ 672.2480; found 672.2514.

### *N*-methyl-*N*-Boc-2-aminoethyl 4,5,7,8,9-penta-*O*-acetyl-3-deoxy-d-*glycero*-α-d-*galacto*-non-2-ulopyranosidonic acid (10)

Compound **9** (3.25 g, 5.00 mmol) was dissolved in pyridine (100 mL), then treated with LiI (13.6 g, 100 mmol, 20 equiv.) and the solution was stirred at 120 °C for 20 h under a N_2_ atmosphere. The mixture was allowed to cool to rt then co-concentrated with PhCH_3_ (3 × 200 mL) and the resulting residue dissolved in CH_2_Cl_2_ (300 mL). The mixture was washed with 1 M HCl (2 × 100 mL), and then with a saturated solution of Na_2_S_2_O_3_ (2 × 100 mL). The combined organic layers were dried (Na_2_SO_4_) and concentrated to a residue that was purified by column chromatography (EtOAc/MeOH 100:0 → 1:1 v/v) to give **10** (1.78 g, 56%, α only) as a pale white amorphous solid. ^1^H NMR (400 MHz, CD_3_OD) δ_H_ 5.39 (ddd, *J* = 9.4, 4.8, 2.5 Hz, 1H, H-8), 5.31 (dd, *J* = 9.4, 2.2 Hz, 1H, H-7), 5.01–4.90 (m, 1H, H-4), 4.86 (m, 1H, H-5), 4.32–4.18 (m, 2H, H-9a, H-6), 4.10 (dd, *J* = 12.5, 4.8 Hz, 1H, H-9b), 3.87 (dt, *J* = 10.4, 5.3 Hz, 1H, -OCH*H*CH_2_NCH_3_COO-), 3.64 (t, *J* = 5.9 Hz, 1H, H-5), 3.53 (dt, *J* = 10.1, 5.7 Hz, 1H, -OC*H*HCH_2_NCH_3_COO-), 3.39 (dd, *J* = 6.4, 5.1 Hz, 2H, -OCH_2_C*H*_2_NCH_3_COO-), 2.91 (s, 3H, -NC*H_3_*), 2.67 (dd, *J*_3eq,3ax_ = 12.8, *J*_3eq,4_ = 4.9 Hz, 1H, H-3_eq_), 2.11 (s, 3H, C=OC*H*_3_), 2.09 (s, 3H, C=OC*H*_3_), 2.00 (s, 3H, C=OC*H*_3_), 1.98 (s, 3H, C=OC*H*_3_), 1.97 (s, 3H, C=OC*H*_3_), 1.81 (t, *J*_3eq,3ax_ = 12.5 Hz, 1H, H-3_ax_), 1.47 (s, 9H, C(C*H*_3_)_3_). ^13^C NMR (101 MHz, CD_3_OD) δ_C_ 172.33 (C=O), 171.59 (C=O), 171.56 (C=O)x2), 170.21 (C-1), 99.88 (C-2), 81.02 (4 °C), 72.38 (C-6), 70.85 (C-4), 69.45 (C-5), 69.17 (C-7), 68.06 (C-8), 63.23 (C-9), 60.99 (-O*C*H_2_CH_2_NCH_3_COO-), 50.06 (-OCH_2_*C*H_2_NCH_3_COO-), 38.79 (C-3), 35.84 (-N*C*H_3_), 28.75, 28.71 (-C(*C*H_3_)_3_), 21.15, 20.71, 20.68, 20.66, 20.59 (-C=O*C*H_3_). HRMS-ESI (m/z) Calcd for C_27_H_41_NO_16_ ([M + Na])^+^ 658.2423; found 658.2304.

### 4-Methylumbelliferyl chloroformate

4-Methylumbelliferone (5.0 g, 28.4 mmol) and triphosgene (5.9 g, 19.9 mmol) were dissolved in dry CH_2_Cl_2_ then cooled to 0 °C under a N_2_ atmosphere. Then a solution of 2 M NaOH (15.6 mL, 31.2 mmol, 1.1 equiv.) was added dropwise and the stirred mixture was allowed to gradual warm to rt over 21 h in an ice/water bath. The white solids were filtered and washed with CH_2_Cl_2_ to give a first crop (3.4 g). The filtrate was concentrated, and the white solids were filtered and wash with CH_2_Cl_2_ to give second crop (5.8 g, 85% combined) of 4-methylumbelliferyl chloroformate as a colorless powder. ^1^H NMR (400 MHz, CDCl_3_) δ_H_ 7.69–7.67 (m, 1H), 7.28–7.21 (m, 2H), 6.34 (s, 1H), 2.47 (s, 3H). The NMR data are in agreement with those reported in the literature (Prost et al. 2014).

### *N*-methyl-(*N*-4′-methylumbelliferylcarbonyl)-2-aminoethyl 4,5,7,8,9-*penta*-*O*-acetyl-3-deoxy-d-*glycero*-α-d-*galacto*-non-2-ulopyranosidonic acid (12)

Protected amine **10** (738 mg, 1.16 mmol) was dissolved in CH_2_Cl_2_ (50 mL) and then treated with TFA (3 mL). The resultant mixture was stirred at rt for 2 hr, and then it was concentrated to a syrupy residue that was co-concentrated three times with PhCH_3_ (50 mL). 4-methylumbelliferyl chloroformate was added to the crude residue containing secondary amine **11** (554 mg, 2.32 mmol, 2 equiv.) and this mixture was dissolved in CH_2_Cl_2_ (30 mL). Following addition of DIPEA (1.2 mL, 6.97 mmol, 6 equiv.) the reaction mixture was stirred at rt under a N_2_ atmosphere for 44 h. The mixture was then diluted with CH_2_Cl_2_ (300 mL) and washed with 1 M HCl (100 mL). The organic layer was dried (Na_2_SO_4_), concentrated, and the resulting residue was purified by column chromatography (EtOAc/MeOH 100:0 → 9:1 v/v) to give **12** (673 mg, 78%) as a colorless amorphous solid (≈1:1 mixture of rotamers). ^1^H NMR (500 MHz, CD_3_OD) δ_H_ 7.79 (d, *J* = 9.1 Hz, 1H, Ar-H), 7.28–7.14 (m, 2H, Ar-H), 6.30 (s, 1H, C=C-*H*), 5.41 (ddd, *J* = 8.5, 5.4, 2.7 Hz, 1H, H-8), 5.30 (ddd, *J* = 8.6, 5.8, 2.0 Hz, 1H, H-7), 5.03 (ddd, *J* = 12.3, 9.3, 4.8 Hz, 1H, H-4), 4.47–4.40 (m, 1H, H-6), 4.36 (dd, *J* = 12.4, 2.6 Hz, 1H, H-9a), 4.28 (dd, *J* = 12.4, 2.6 Hz, 1H, H-9a), 4.10 (dd, *J* = 17.1, 5.4 Hz, 1H, H-9b), 4.06–4.01 (m, 1H, H-9b), 4.00–3.94 (m, 1H, -OCH*H*CH_2_NCH_3_COO-), 3.72 (dd, *J* = 10.6, 5.4 Hz, 1H, -OCH_2_CH*H*NCH_3_COO-), 3.67–3.63 (m, 1H, OC*H*HCH_2_NCH_3_COO-), 3.55 (d, *J* = 2.8 Hz, 1H, -OCH_2_C*H*HNCH_3_COO-), 3.22–3.10 (2 s, 3H, -NC*H_3_*), 2.70 (dd, *J*_3eq,3ax_ = 12.2, *J*_3eq,4_ = 6.1 Hz, 1H, H-3_eq_), 2.48 (d, *J* = 1.3 Hz, 3H, Ar-C*H*_3_), 2.12 (s, 3H), 2.08 (s, 3H, C=OC*H*_3_), 1.99 (s, 3H, C=OC*H*_3_), 1.97 (s, 3H, C=OC*H*_3_), 1.95 (s, 3H, C=OC*H*_3_), 1.77 (t, *J* = 12.3 Hz, 1H, H-3_ax_), 1.68 (t, *J*_3eq,3ax_ = 12.3 Hz, 1H, H-3_ax_). ^13^C NMR (101 MHz, CD_3_OD) δ_C_ 172.39, 171.88 (C=O), 171.76, 171.70 (C-1), 162.70, 155.76, 155.67, 155.31, 155.02 (C=O), 126.94, 119.59, 118.56, 114.54, 114.48, 111.18, 111.06 (Ar-C), 101.01 (C-2), 72.17, 71.45 (C-4), 69.95(C-7), 69.63 (C-8), 68.48 (C-6), 64.04 (-O*C*H_2_CH_2_NCH_3_COO-), 63.43 (-OCH_2_*C*H_2_NCH_3_COO-), 50.61 (C-9), 39.29, 39.08 (C-3), 36.43, 36.32 (-N*C*H_3_), 21.30, 20.76, 20.70, 20.61 (-C=O*C*H_3_), 18.66 (Ar-*C*H_3_). HRMS-ESI (m/z) Calcd for C_33_H_39_NO_18_ ([M + Na])^+^ 760.2065; found 760.2035.

### *N*-methyl-(*N*-4′-methylumbelliferylcarbonyl)-2-aminoethyl 3-deoxy-d-*glycero*-α-d-*galacto*-non-2-ulopyranosidonic acid (4)

Protected Kdn glycoside **12** (421 mg, 0.57 mmol) was dissolved in MeOH (20 mL) and treated with K_2_CO_3_ (79 mg, 0.57 mmol, 1 equiv.). The resulting mixture was stirred at rt for 14 h. An additional portion of K_2_CO_3_ (40 mg, 0.28 mmol, 0.5 equiv.) was added and stirring was continued at rt for 8 h. Following completion of the deprotection the reaction mixture was concentrated and the residue was purified by column chromatography (EtOAc/MeOH 6:4) to obtain Kdn-SICL-4MU **4** (228 mg, 76%) as a white solid. (~1:2 mixture of rotamers) ^1^H NMR (400 MHz, CD_3_OD) δ_H_ 7.79 (dd, *J* = 9.4, 1.7 Hz, 1H, Ar-H), 7.24–7.16 (m, 2H, Ar-H), 6.30 (d, *J* = 1.4 Hz, 1H, C=C-*H*), 4.09–3.93 (m, 2H, -OCH*H*CH_2_NCH_3_COO-, -OCH_2_CH*H*NCH_3_COO-), 3.91–3.77 (m, 3H, H-7, H-8, H9a), 3.78–3.54 (m, 5H, -OC*H*HCH_2_NCH_3_COO-, -OCH_2_C*H*HNCH_3_COO-, H-4, H-6, H9b), 3.53–3.41 (m, 1H, H-5), 3.13 (2 s, 3H, -NC*H_3_*), 2.79 (dd, *J*_3eq,3ax_ = 12.2 Hz, *J*_3eq,4_ = 4.9 Hz, 1H, H-3_eq_), 2.49 (d, *J* = 1.3 Hz, 3H, Ar-C*H*_3_), 1.65–1.49 (m, 1H, H-3_ax_). ^13^C NMR (151 MHz, D_2_O) δ_C_ 173.61, 173.45 (C-1), 163.81 (C=O), 155.69(C=O), 155.65 (C=O), 153.37, 153.32, 152.88, 126.24, 126.14, 118.91, 118.68 (Ar-C), 117.43(4^°^ Ar), 112.63(C=*C*H), 110.05, 109.88 (Ar-C), 100.55, 100.49 (C-2), 73.50 (C-6), 71.98, 71.95 (C-4), 70.21 (C-7), 69.84 (C-8), 67.68, 67.63 (C-6), 62.51, 62.39 (-O*C*H_2_CH_2_NCH_3_COO-), 61.84, 61.39 (-OCH_2_*C*H_2_NCH_3_COO-), 48.99, 48.94 (C-9), 39.91, 39.79 (C-3), 35.19 (-N*C*H_3_), 17.93 (Ar-*C*H_3_). HRMS-ESI (m/z) Calcd for C_23_H_29_NO_11_ ([M + Na])^+^ 550.1537; found 550.1525.

## Conclusions

We have developed a self-immolative Kdn glycoside (**4**) in which the liberated aglycon, after enzymatic cleavage, undergoes a facile base-promoted cyclization to release the fluorescent aryloxide of 4-methylumbelliferone. Using this compound we optimized a 384-well assay in order to perform high-throughput screens. The results from these screens allowed use to identify two competitive inhibitors of the Kdnase from *A. fumigatus* (*Af*), which is the leading cause of invasive aspergillosis (IA). Given that the *A. fumigatus* Kdnase (*Af*K) plays a critical, but unknown, role in cell wall integrity and virulence, our hit compounds are promising starting points for inhibitor optimization, which for lithospermic acid would benefit from starting with attempts to remove its the catechol moieties.

This strategy has great potential for Kdnases, and it can be extended to address the challenges posed by the use of activated oct- and non-2-ulosonic acid glycosides, such as those from Kdo, *N-*acetylneuraminic acid, of the corresponding glycoside hydrolases. For example, an analogous sialic acid glycoside could be used for screening inhibitors of neuraminidases from human (NEU1, GH33) (Guo et al. 2018), bacterial (GH33, GH156, and GH181) (Chuzel et al. 2018; Shuoker et al. 2023), and viral (GH34, GH58, and GH83) (Pelkonen et al. 1989; Taylor 1996; Crennell et al. 2000; Sun 2021) origins. This study demonstrates an efficient synthesis and screening approach for a Kdnase glycoside that effectively addresses intrinsic reactivity issues associated with activated glycosides to provide a robust and sensitive HTS assay. Collectively, these findings have resulted in the discovery of new competitive inhibitors for Kdnases.

## Supplementary Material

Supporting_Information_cwae094

## Data Availability

The data underlying this article will be shared upon reasonable request to the corresponding author. Email: bennet@sfu.ca.
