# Supplementary material for: A self-immolative Kdn-glycoside substrate enables high-throughput screening for inhibitors of Kdnases
Source: Glycobiology. 2024 Nov 21;35(1):cwae094. doi: 10.1093/glycob/cwae094 (PMC11727334; doi:10.1093/glycob/cwae094)
Supplement: Supporting_Information_cwae094 [file supporting_information_cwae094.pdf]

Supporting Information

A Self-immolative Kdn-glycoside substrate enables high-throughput screening for inhibitors of Kdnases.

A. Nejatie,[1] C. Proceviat,[1] Christina Gros,[1] Elizabeth Steves,[2] M.M. Moore,[2] D.J. Vocadlo,[1,3] A.J. Bennet[1]*

[1] Department of Chemistry, Simon Fraser University, 8888 University Drive, Burnaby, BC, V5A 1S6, Canada; [2] Department of Biological Sciences, Simon Fraser University, 8888 University Drive, Burnaby, BC, V5A 1S6, Canada. [3] Department of Molecular Biology and Biochemistry, Simon Fraser University, 8888 University Drive, Burnaby, BC, V5A 1S6, Canada.

*E-mail: bennet@sfu.ca

Table of Contents

Enzymatic rate constants for the hydrolysis of **4** (Table S1)	S2
Table of inhibition values of Kdn2en, **8**, **9**, and **12** against *Af*K (Table S2)	S2
DSF temperature shifts for Kdnases with inhibitors (Tables (S3 and S4)	S3
Synthetic schemes S1–S3	S4
Stacked NMR spectra showing *Af*K-catalyzed hydrolysis of **4** (Figure S1)	S5
Cyclization rate of carbamate **6** (Figure S2)	S6
Correlation of enzyme activity vs concentration (Figure S3)	S6
Calibration curve for 4MU fluorescence versus concentration (Figure S4)	S7
Michaelis–Menten plot for the *Af*K-catalyzed hydrolysis of **4** (Figure S5)	S7
*Af*K activity assay in the presence of Tween 20 (Figure S6)	S8
Stability of fluorescence signal as a function of time (Figure S7)	S8
Inhibition of *Af*K by **13** and **14** (Figure S8)	S9
Check for time dependant inhibition of *Af*K by **13** (Figure S9)	S10
IC_{50} of caffeic acid, a monomer of **8** against *Af*K (Figure S10)	S10
Inhibition of *At*K, *Tr*K, and *Mv*NA by **13** (Figure S11)	S11
A summary of the HTS using the Chembridge library against *Af*K (Figure S12)	S12
Inhibition of *Af*K, *At*K, *Tr*K, and *Mv*NA by **17** (Figure S13)	S12
Thermal shift assay of *Af*K, *At*K, and *Tr*K with **2**, **13**, and **17** (Figure S14)	S13
NMR Spectra (Figures S15-S22)	S14–S21

Table S1. Kinetic parameters for the enzyme-catalyzed hydrolysis of probes Kdn-4MU and Kdn-SICL-4MU. Reactions were run in 100 mM NaOAc at pH 4 containing 0.01% BSA, at 21 °C. The stop solution contained 200 mM K_2CO_3 pH 12.

**Substrate**	**K_m (mM)**	**k_{cat} (s^{-1})**	**k_{cat}/K_m ($M^{-1}s^{-1}$)**
Kdn-4MU[a]	0.23 ± 0.02	(41 ± 4)	$(1.8 \pm 0.1) \times 10^5$
Kdn-SICL-4MU (**4**)	0.95 ± 14	3.7 ± 0.2	$(4.0 \pm 0.8) \times 10^3$

[a] values taken from Telford et al., *J Biol Chem*, **2011**, 10783.

Table S2. Measured IC_{50} and Hill values for inhibition of *Af*K by Kdn2en (**2**), **13**, **14**, and **17**using Kdn4MU as a substrate in sodium acetate buffer (100 mM, pH 4.0) containing 0.01% BSA at 37 °C. K_i values were calculated using the equation $K_i = IC_{50}/([S]/(K_m+1))$.

Compound	IC_{50} (µM)	K_i (µM)	Hill
Kdn2en (**2**)[a]	556	102	–
**13**[b]	22.8 ± 1.0	19.5	–1.32
**14**[b]	65.8 ± 1.7	58.4	–0.92
**17**[b]	115.3 ± 2.0	46.5	–0.81

[a] values taken from Telford et al. (3) [b] Values from this work.

Table S3. Differential scanning fluorometry-based (DSF) assay for inhibitors **2**, **13**, and **17** binding to *Af*, *At*, *Tr and Mv*NA. Protein thermal shifts(ΔT_m) using DMSO (2.5%), and inhibitors **2**, **13**, and **17** at 500 µM against the Kdnase of *Af*, *At*, *Tr and Mv*NA. Assay done using 5–10 µM enzyme and 100 mM pH 4.0 NaOAc (pH 5.5 *Mv*NA). A temperature range of 25°C to 95°C was used with a gradient of 0.05°C/sec.

Enzyme	DMSO (°C)	**2** (°C)	**13** (°C)	**17** (°C)
A. fumigatus	51.5 ± 0.4	53.2 ± 0.1	51.4 ± 0.1	54.6 ± 0.1
A. terreus	51.9 ± 0.3	55.3 ± 0.1	51.1 ± 0.1	55.4 ± 0.1
T. rubrum	34.7 ± 0.1	49.7 ± 0.2	48.4 ± 0.1	50.9 ± 0.1
M. viridifaciens	53.9 ± 0.1	54.4 ± 0.1	54.9 ± 0.4	59.1 ± 0.1

Table S4. Thermal shifts (ΔT_m) for stabilization of *Af*K, *At*K, *Tr*K *and Mv*NA on inhibitor binding relative to the DMSO (2.5%) control.

Enzyme	**2** (°C)	**13** (°C)	**17** (°C)
A. fumigatus	+1.7 ± 0.1	–0.1 ± 0.1	+3.1 ± 0.1
A. terreus	+3.4 ± 0.1	–0.8 ± 0.1	+3.5 ± 0.1
T. rubrum	+15.0 ± 0.2	+13.7 ± 0.1	+16.2 ± 0.1
M. viridifaciens	+0.5 ± 0.1	+1.0 ± 0.4	+5.2 ± 0.1

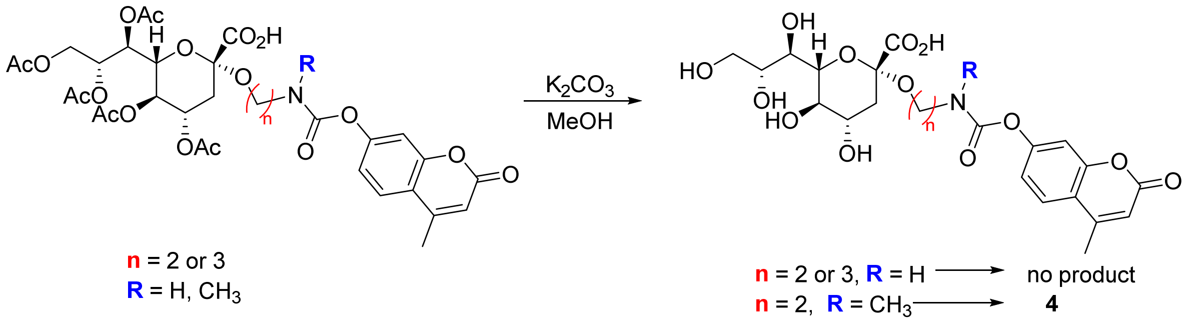

Scheme S1. Reactivity of linkers containing a N-H carbamate. The substrates with R = H were labile under mildly basic conditions, which gave an E1CB elimination and free fluorophore. The tertiary carbamate (R = CH_3) was stable to the basic deprotection conditions to result in the production of glycoside **4**.

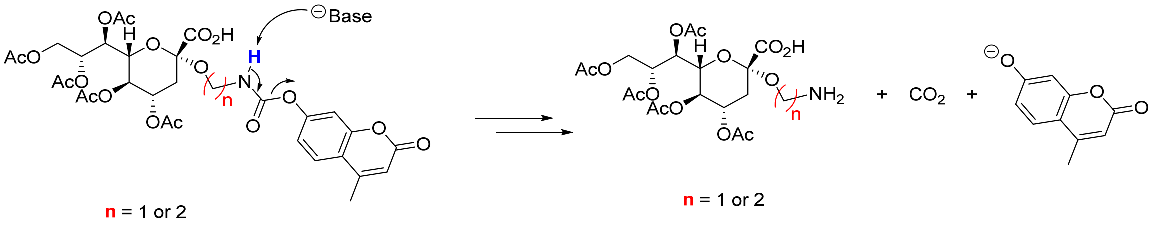

Scheme S2. E1CB mechanism for N-H carbamate decomposition in the presence of base.

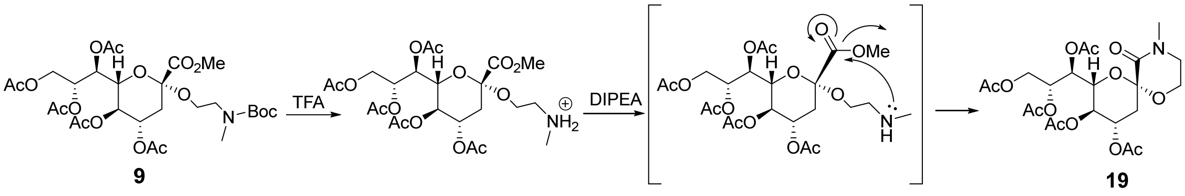

Scheme S3. Intramolecular cyclization of Kdn alkylamino linker intermediate in the presence of base resulting in formation of lactam **19**.

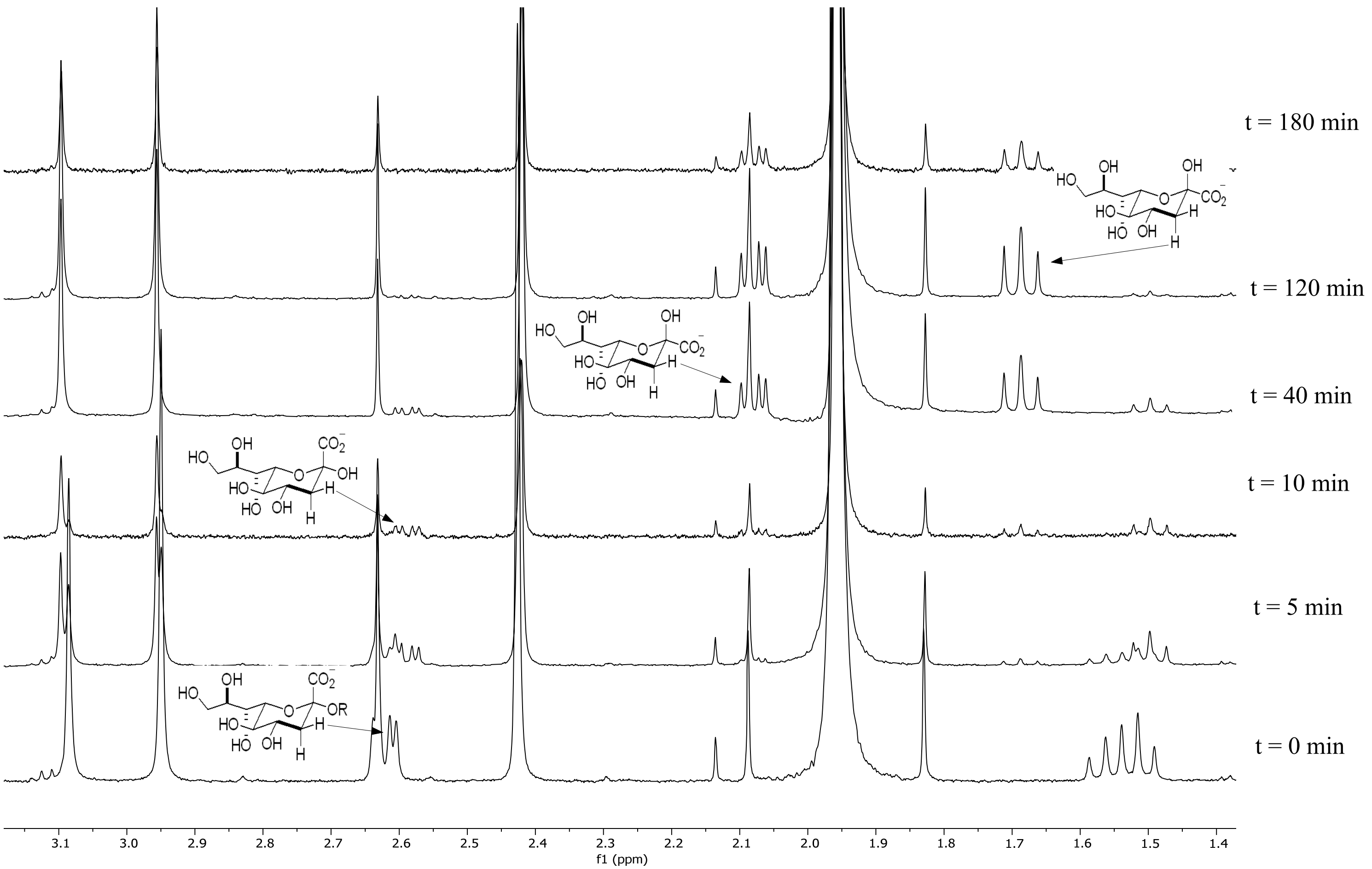

Figure S1. Stacked plot of ^{1}H NMR spectra for the hydrolysis of **4** in buffer after the addition of *Af*K at t = 0 min. Reaction was performed in 100 mM NaOAc pH 4 containing 0.01% BSA, at 21 °C. Arrows show the assignments for the C3-protons of the substrate and product Kdn.

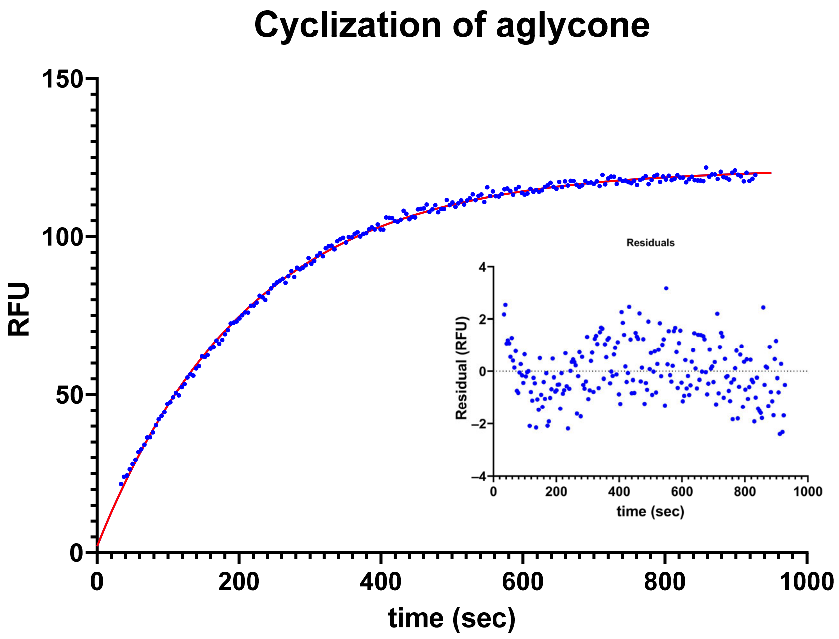

Figure S2. Production of fluorescent signal following the addition of stop solution. Data points in blue, fit to a standard first-order rate equation is shown by the red line [$k_{obs} = (4.68 \pm 0.03) \times 10^{-3}$ s^{-1}]. Insert is the fitted residuals (data – curve fit).

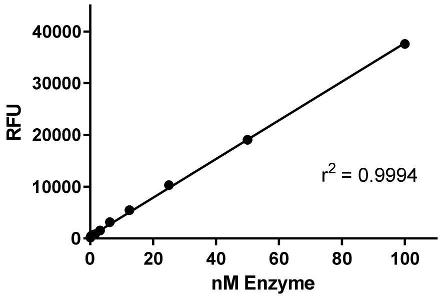

Figure S3. Linear correlation in enzymatic activity. All enzyme concentrations ranging from 0.1–100 nM with the probe **4** ranging from 50–500 µM displayed linear fits. Plot shown is the hydrolysis of **4** (100 µM) catalyzed by [*Af*K] (0.1–100 nM) with samples taken at 10 minutes, and the signal produced after addition of the basic (100 mM pH 12 K_2CO_3) stop solution.

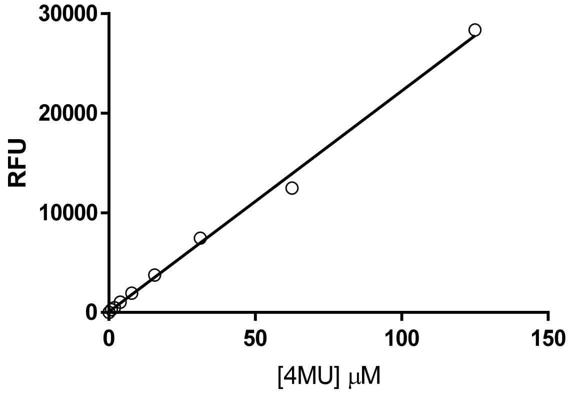

Figure S4. Calibration curve for 4-methylumbelliferone anion. Solutions were made in sodium acetate buffer (100 mM, pH 4.0) containing 0.01% BSA with the concentration range for 4-methylumbellerone being 0.24–125 μM at 37 °C. Fluorescence readings were taken after the addition of the basic (100 mM pH 12 K_2CO_3) stop solution.

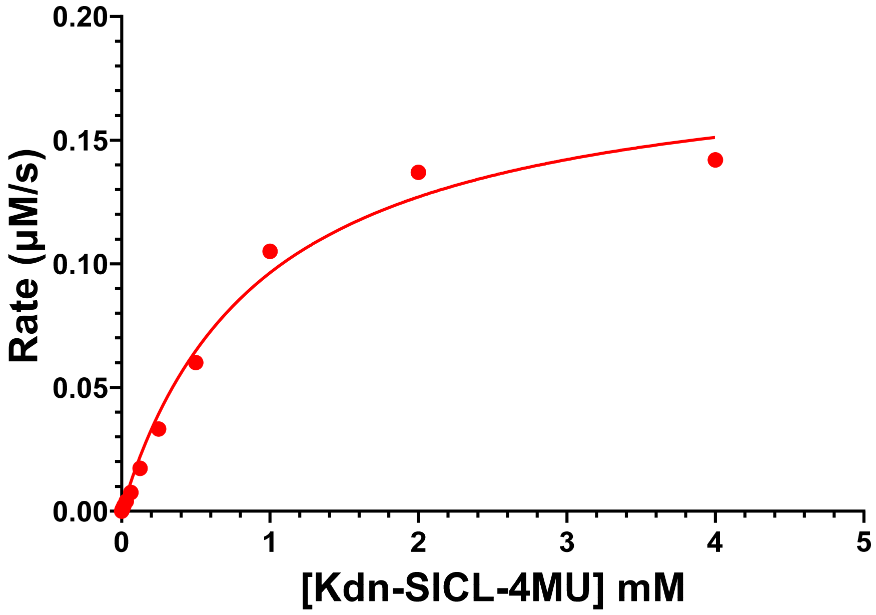

Figure S5. Michaelis-Menten plot for the *AfK*-catalyzed hydrolysis of **4**. Conditions: sodium acetate buffer (100 mM, pH 4.0) containing 0.01% BSA, at 37 °C, [*Af*K] = 100 nM. For each substrate concentration, a total of twelve time points were measure following addition of basic stop solution (100 mM pH 12 K_2CO_3) in ten-minute intervals to give the initial rate measurements.

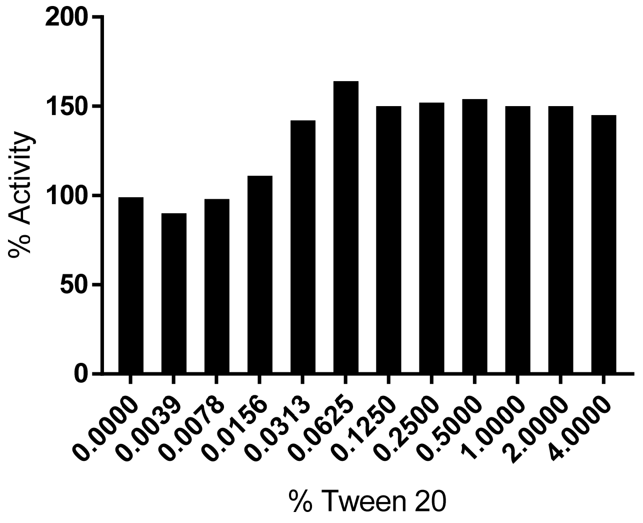

Figure S6. *Af*K activity in the presence of different Tween 20 concentrations.

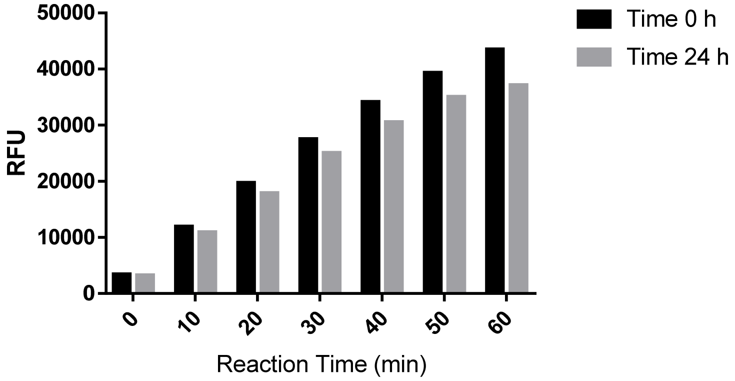

Figure S7. Stability of fluorescence signal as a function of time. A minimal decrease in signal intensity (RFU) occurs during 24 hrs at room temperature.

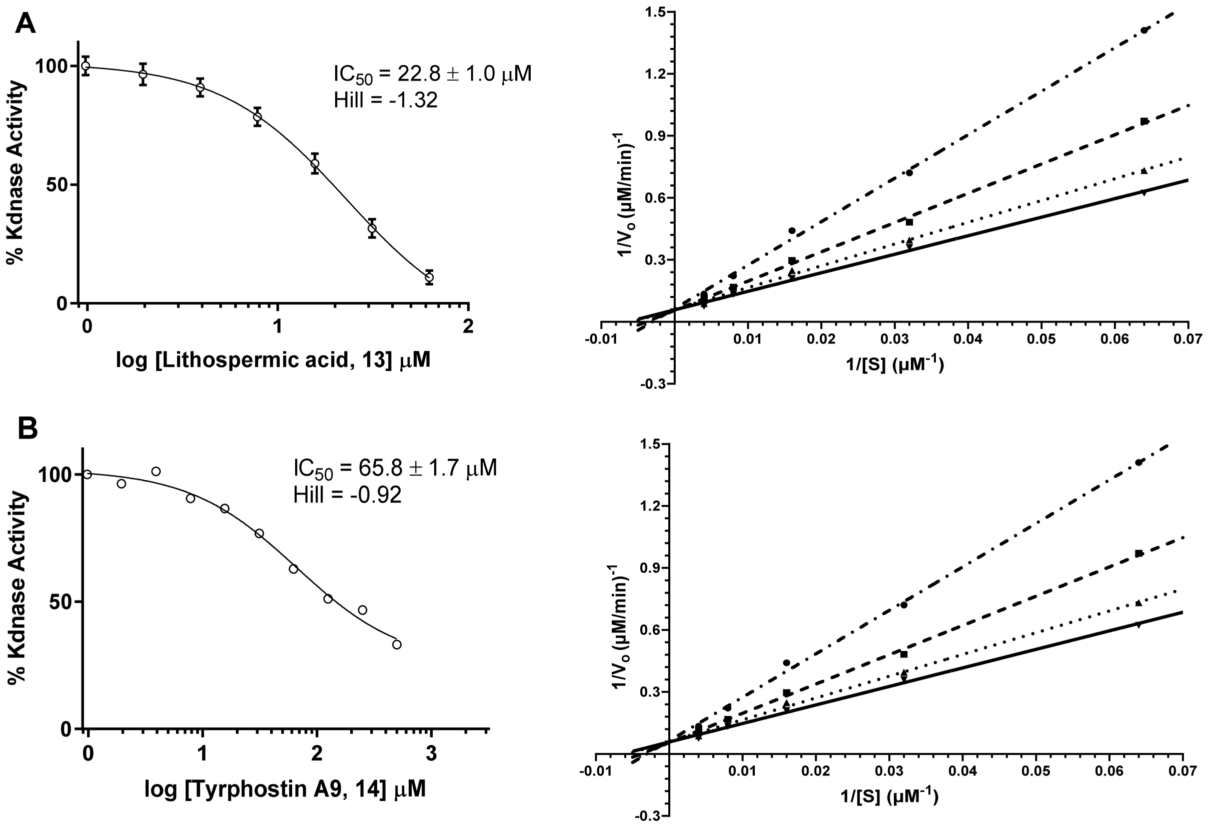

Figure S8. Inhibition of *Af*K by compounds **13** and **14**. A) IC_{50} plot (left) and Lineweaver-Burk plot for the inhibition of *Af*K by Lithospermic acid **13**; concentrations of are 62.5 μM (dash-dot), 31.25 μM (dash), 6.8 μM (dot), and 3.9 μM (solid), and B) IC_{50} plot and Lineweaver-Burk plot for the inhibition of *Af*K by Tyrphostin A9 **14**.; concentrations of are 250 μM (dash-dot), 125 μM (dash), 62 μM (dot), and 31 μM (solid).

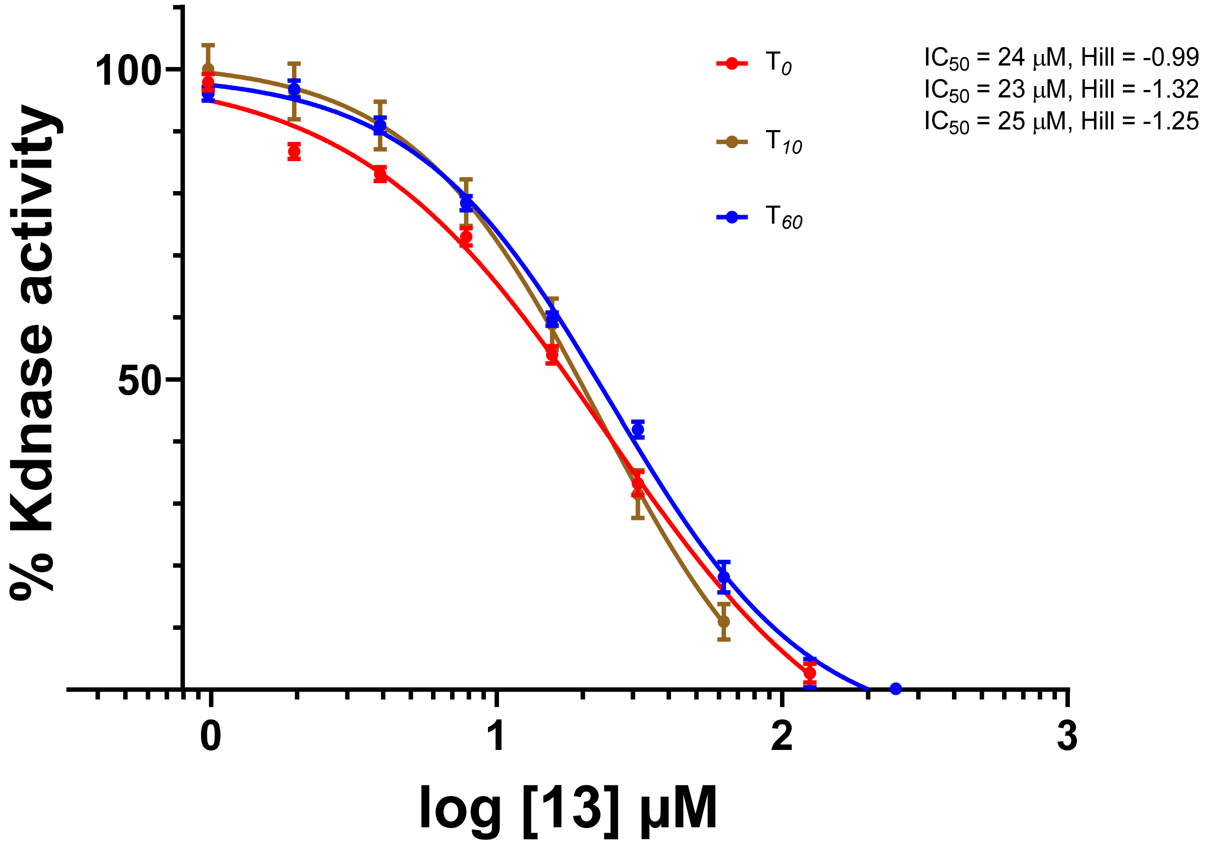

Figure S9. Measurement of IC_{50} values for the inhibition of *Af*K by Lithospermic acid **13**. Time points are 0 (red), 10 (brown) and 60 (blue) mins. Inhibitor concentrations ranged from 0.2–500 μM **13** with 50 μM Kdn4MU **1** substrate.

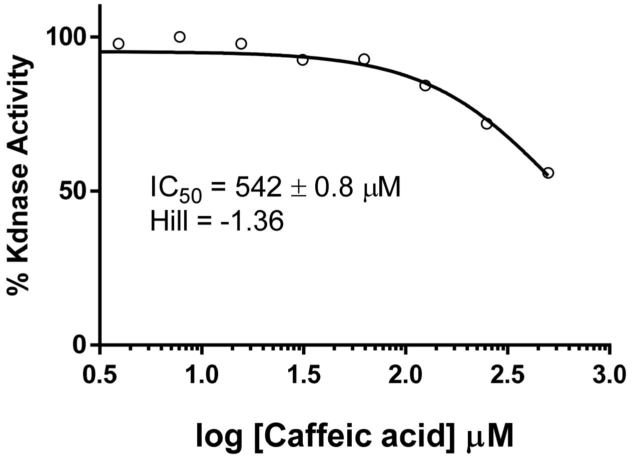

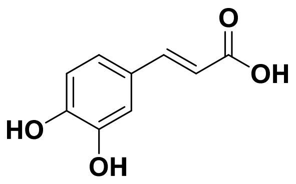

20

Figure S10. IC_{50} of caffeic acid (**20**), a monomer of Lithospermic acid, against *Af*K.

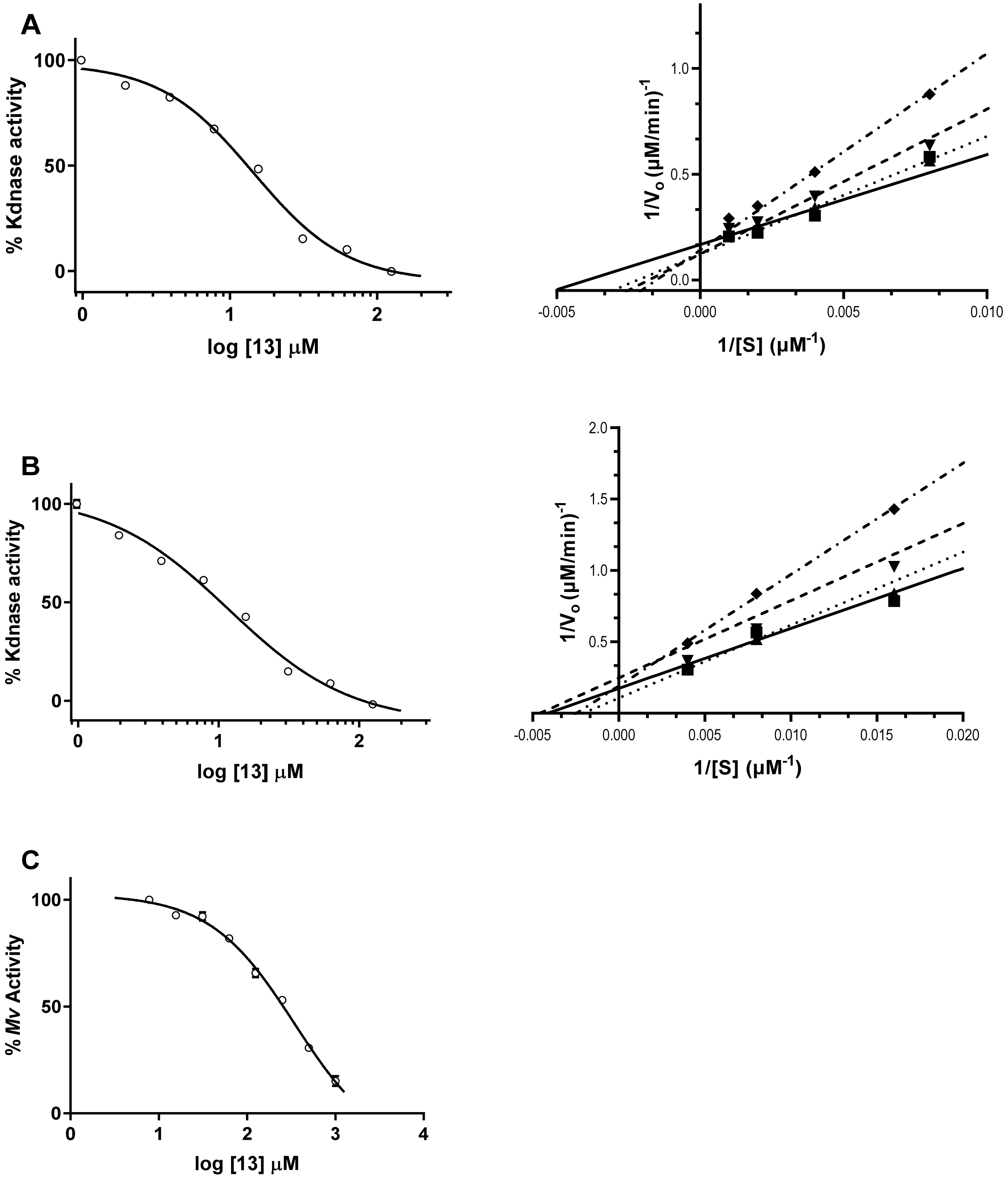

Figure S11. Inhibition of Kdnases from *At* and *Tr* by Lithospermic acid **13**. IC_{50} (left) and Lineweaver-Burk Plot (right) of **13** against the Kdnase of (A) *At;* concentrations are 15.63 μM (dash-dot), 7.81 μM (dash), 3.91 μM (dot), and 1.95 μM (solid). (B) *Tr;* concentrations are 15.63 μM (dash-dot), 7.81 μM (dash), 3.91 μM (dot), and 1.95 μM (solid). (C) IC_{50} of *Mv* by Lithospermic acid **13**.

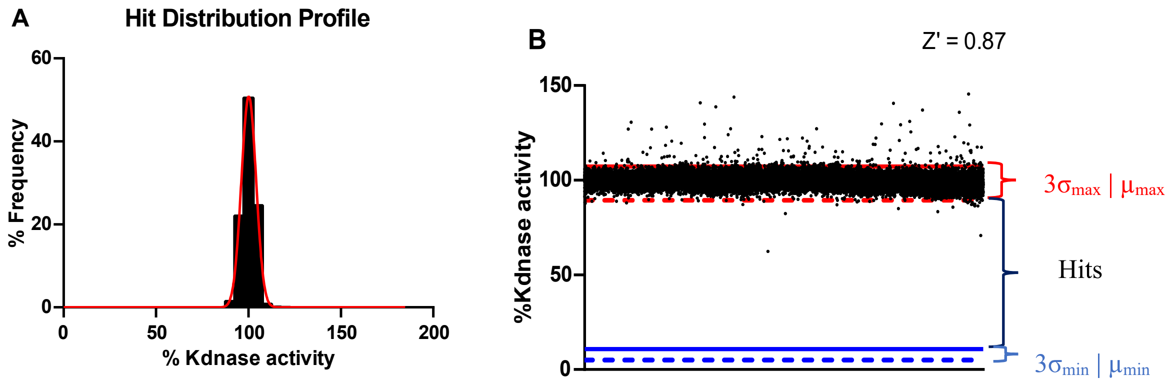

Figure S12. A summary screening of Chembridge library against *Af*K. (A) Hit distribution profile representing residual Kdnase activity from the screen. (B) Screening results of 23,040 known bioactive compounds. μ_{max} represents the mean signal of uninhibited positive control. μ_{min} represents the mean signal of the negative control in the absence of enzyme.

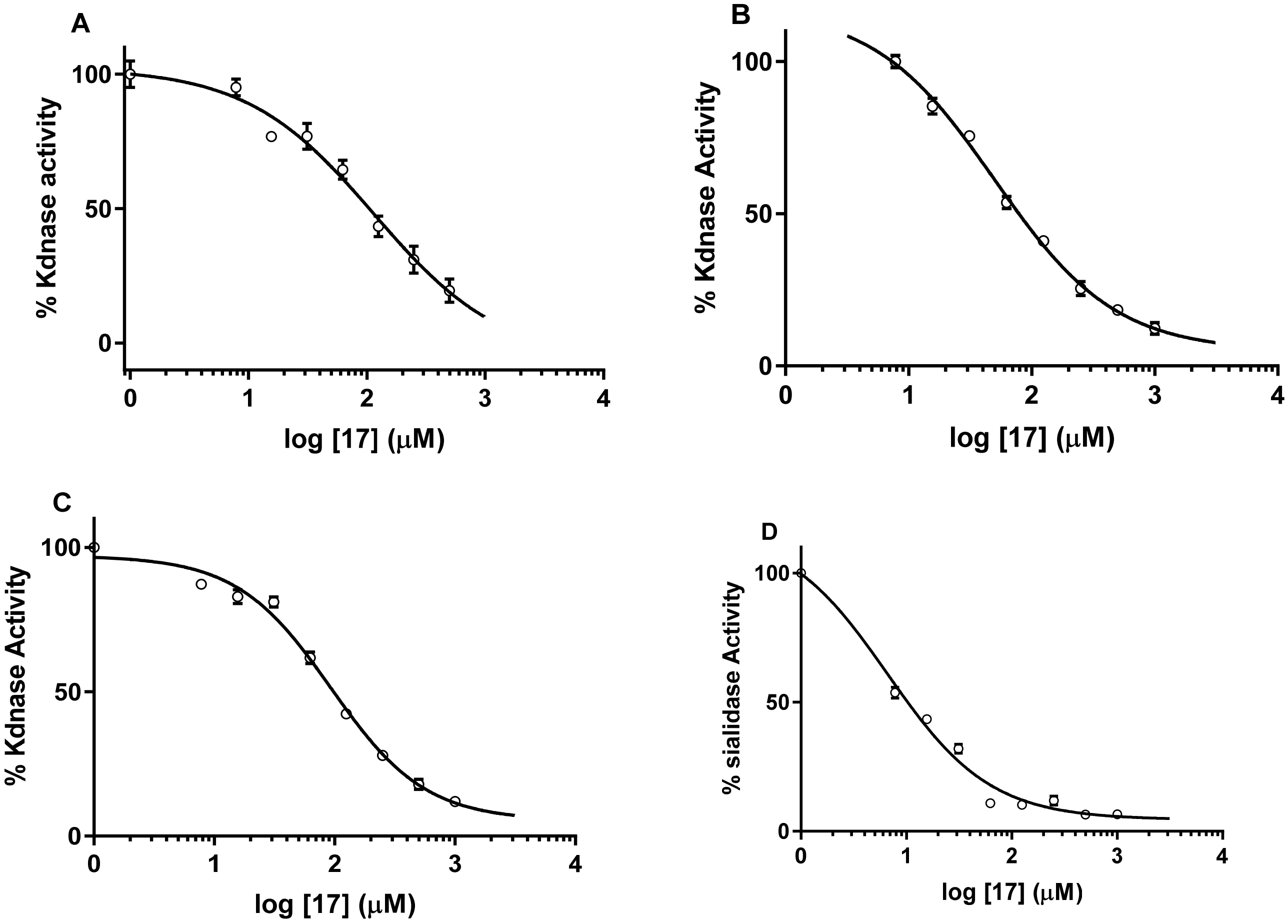

Figure S13. Inhibition of sialidases *Af*, *At*, *Tr* and *Mv* by compound **17**. IC_{50} of **17** against the Kdnase of *Af* (A), *At* (B), *Tr* (C) and *Mv*NA (D).

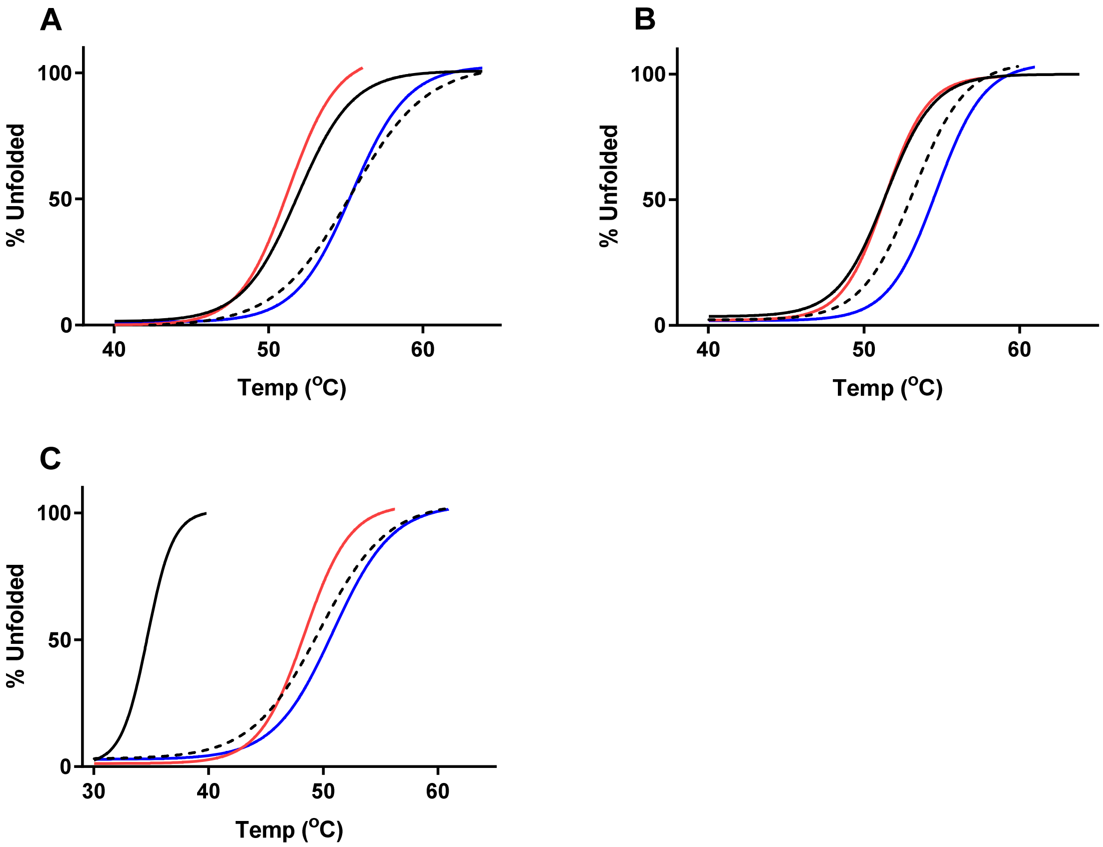

Figure S14. Differential scanning fluorometric-based thermal shift assay showing thermal stabilization of *Af*K (A, 5 μM), *At*K (B, 5 μM), and *Tr*K (C, 500 nM). DMSO was used as a negative control (black), **2** as a positive control (dash). Inhibitors **13** (red) stabilizes neither *Af*K nor *At*K but does stabilize *Tr*K. Inhibitor **17** (blue) stabilizes all three Kdnases.

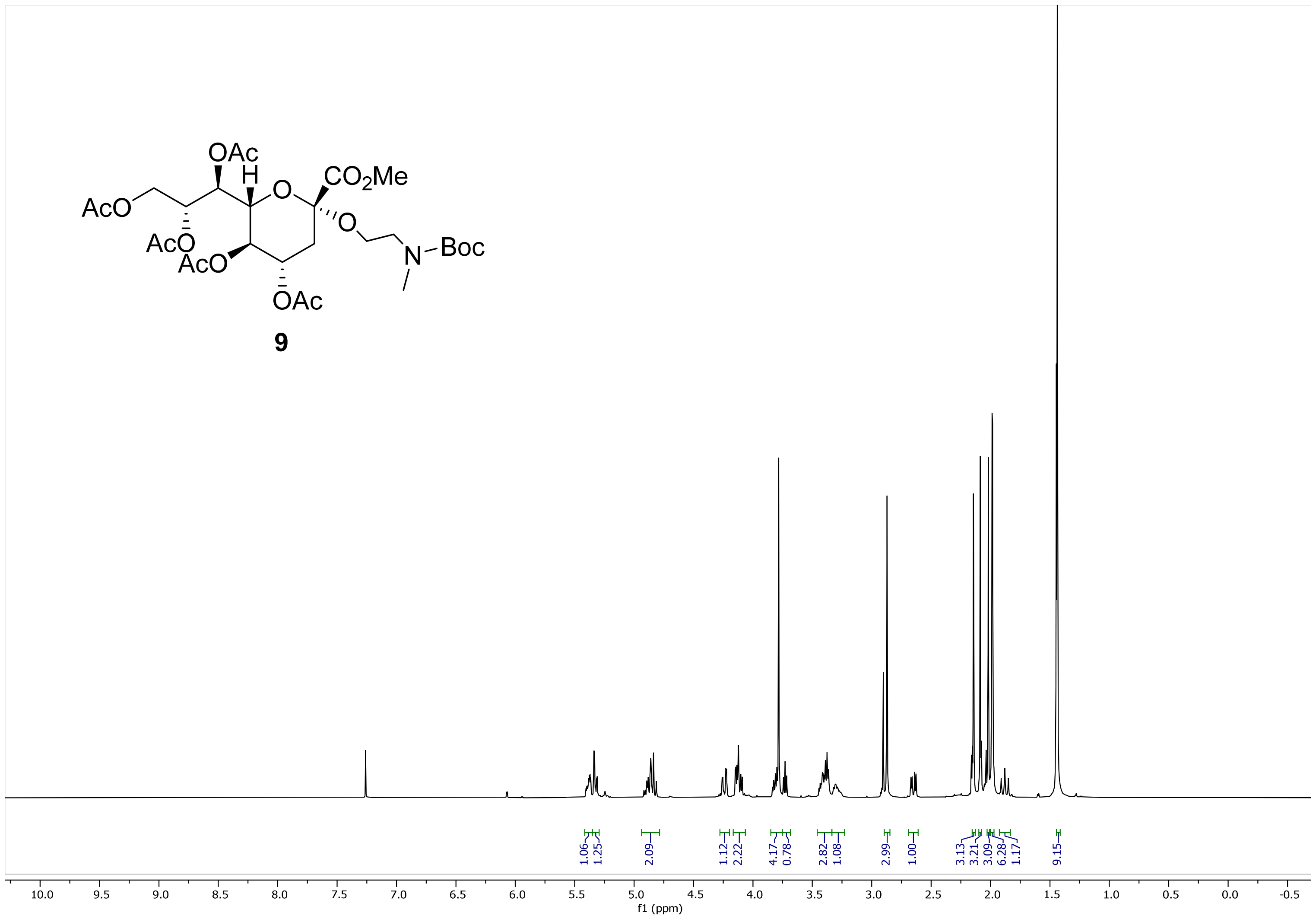

Figure S15. ^{1}H-NMR spectrum of protected Kdn glycoside **9** in $CDCl_3$ (400 MHz).

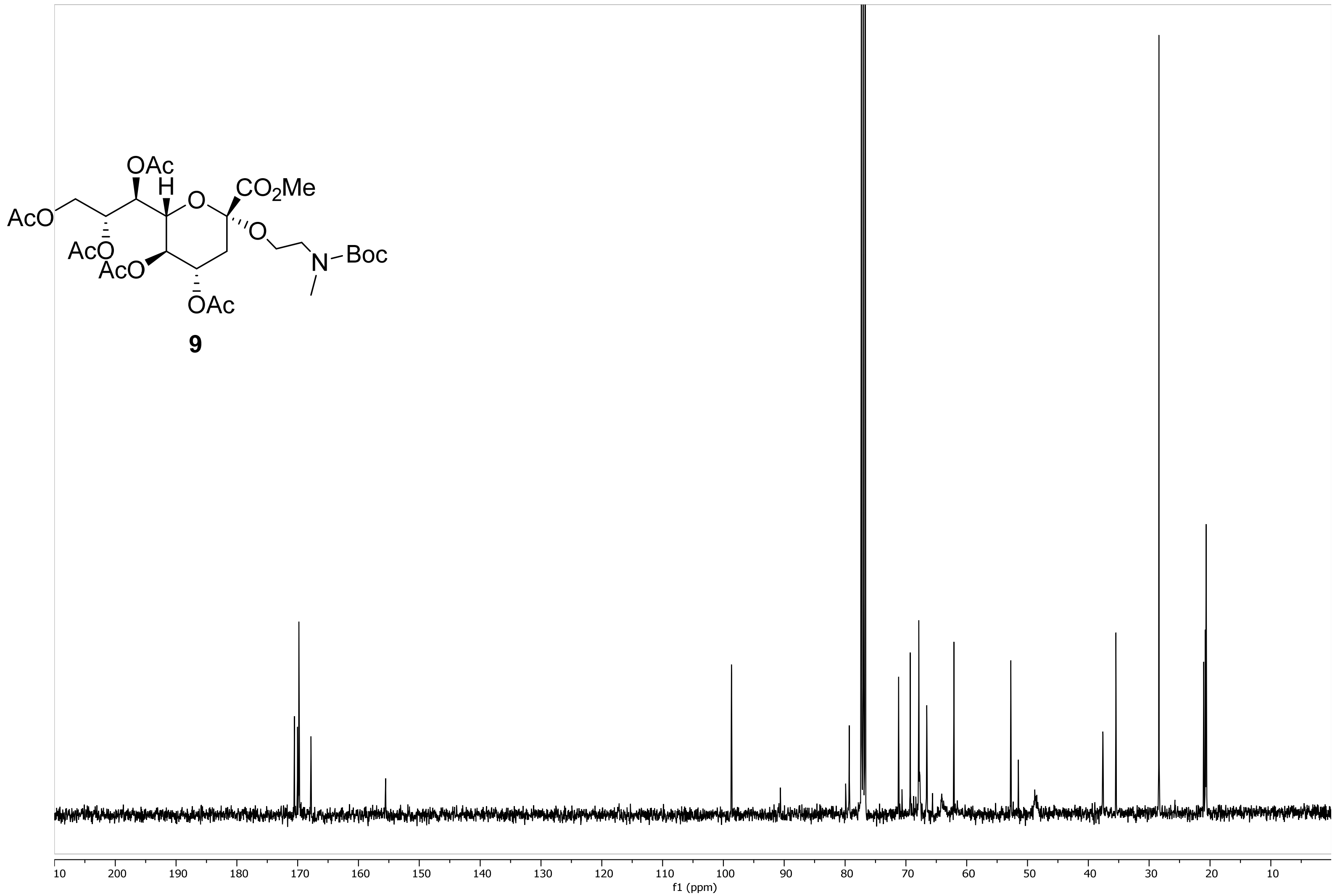

Figure S16. ^{13}C ^{1}HNMR spectrum of protected Kdn glycoside **9** in $CDCl_3$ (101 MHz).

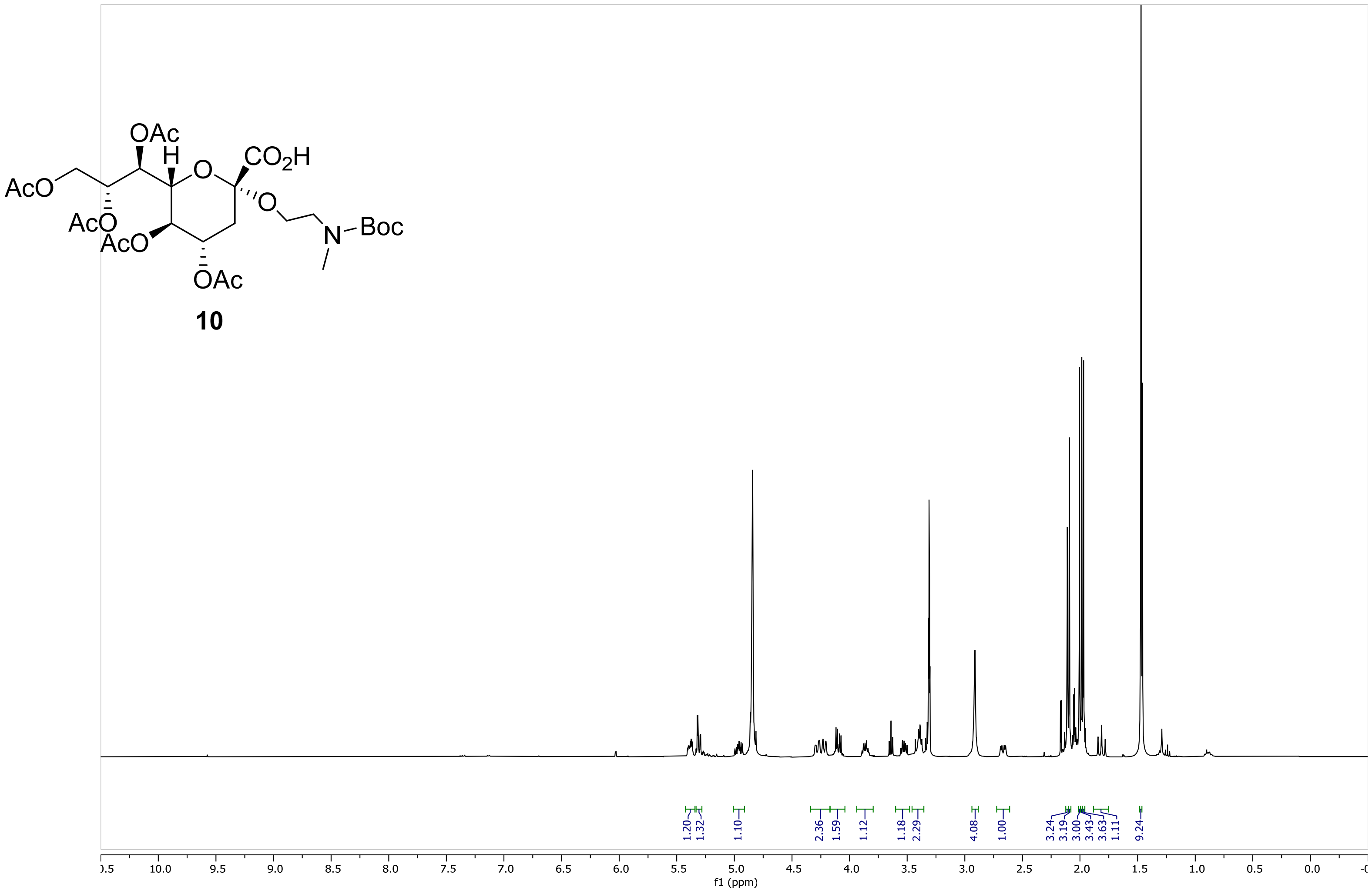

Figure S17. ^{1}H NMR spectrum of protected Kdn glycoside **10** in CD_3OH (400 MHz).

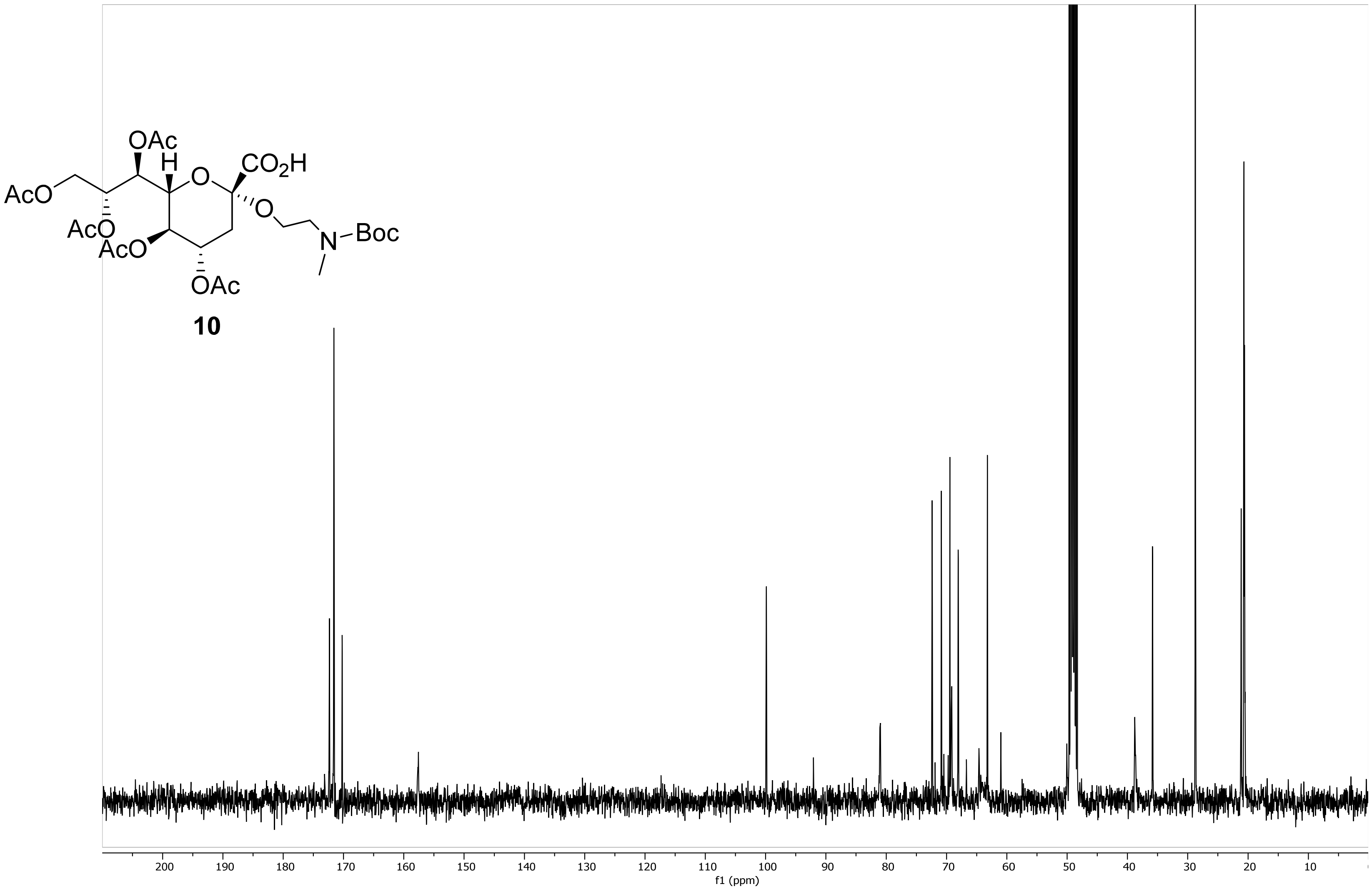

Figure S18. ^{13}C ^{1}HNMR spectrum of protected Kdn glycoside **10** in CD_3OH (101 MHz).

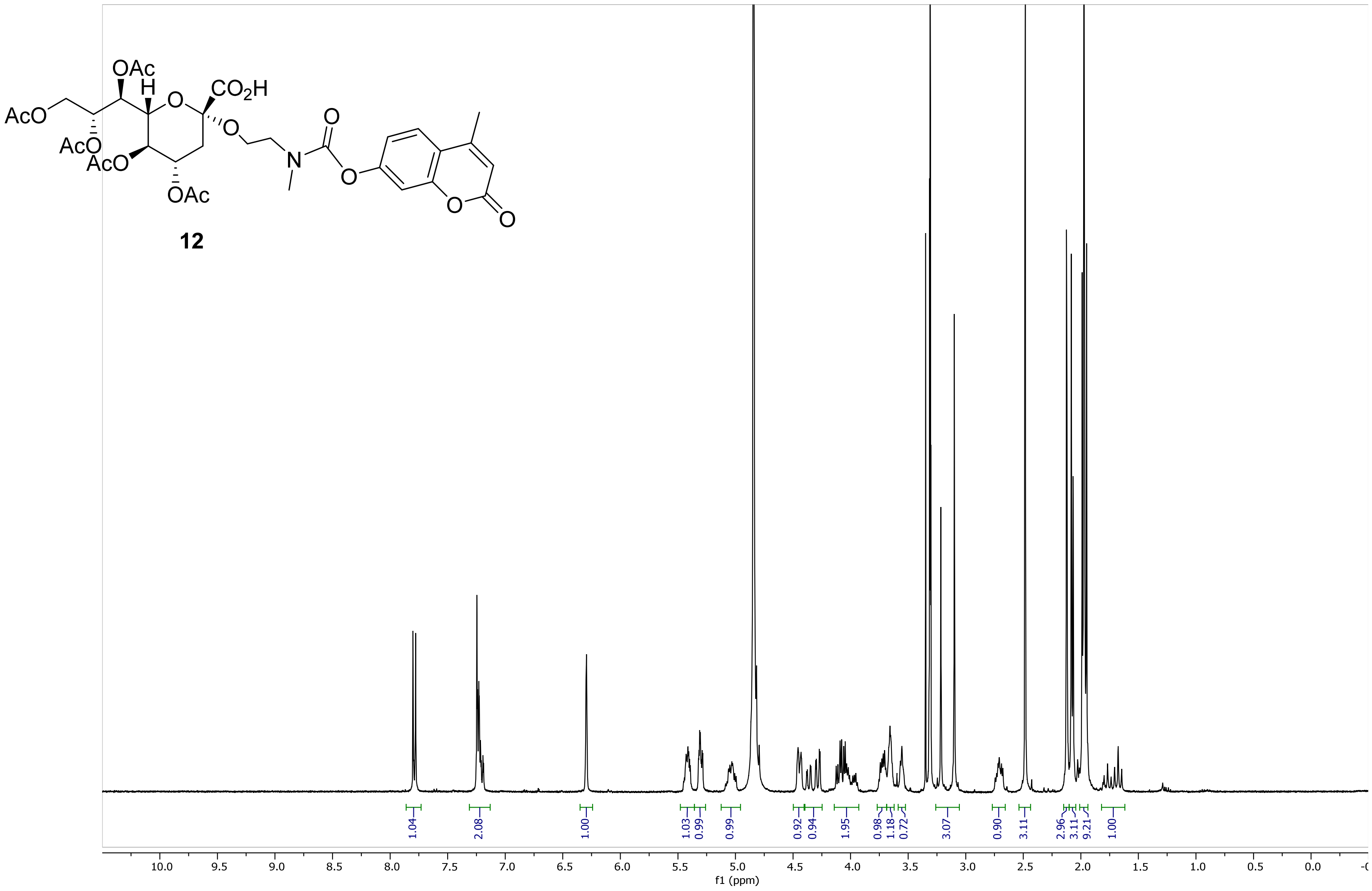

Figure S19. ^{1}H NMR spectrum of protected Kdn glycoside **12** in CD_3OH (400 MHz).

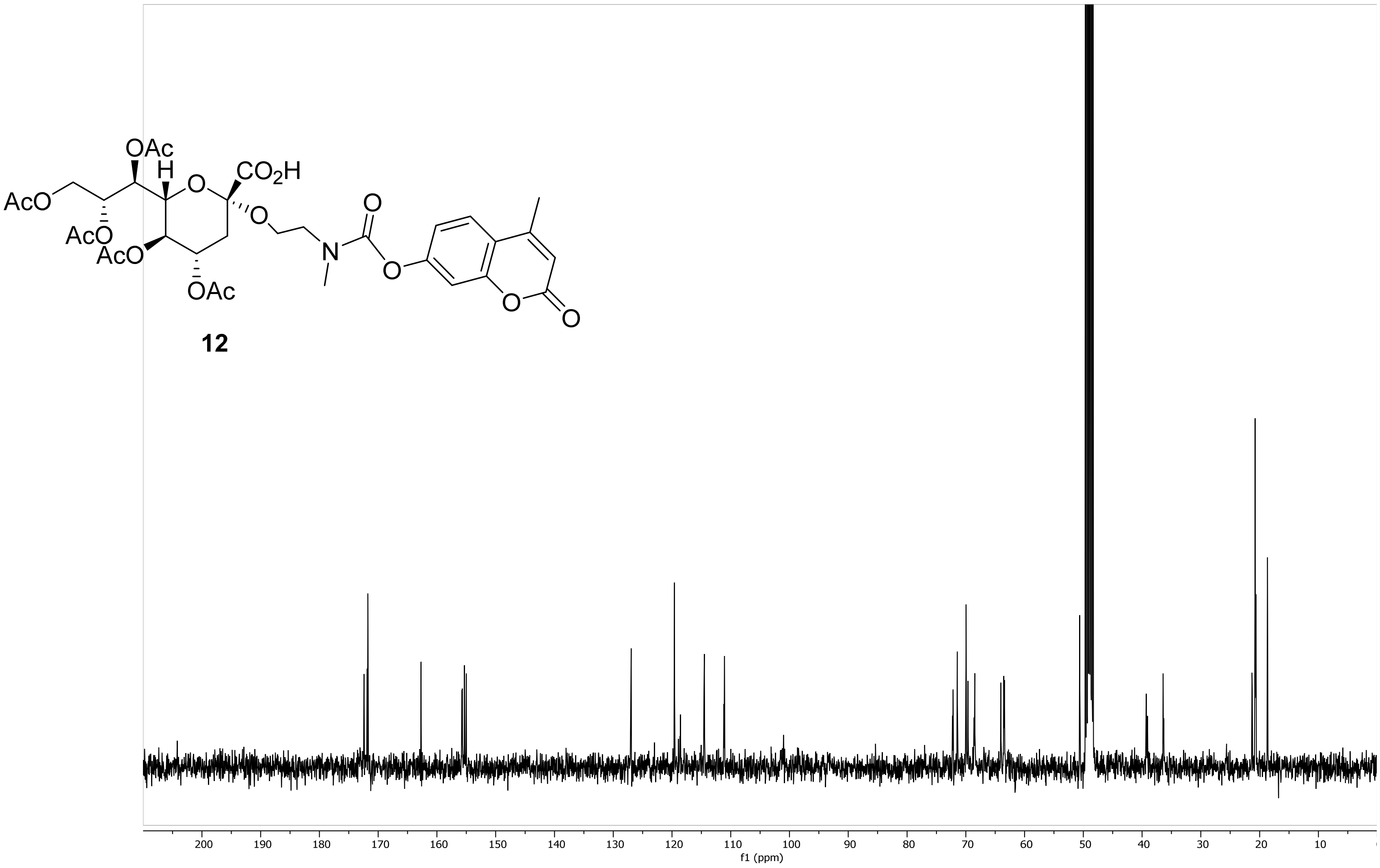

Figure S20. ^{13}C ^{1}HNMR spectrum of protected Kdn glycoside **12** in CD_3OH (101 MHz).

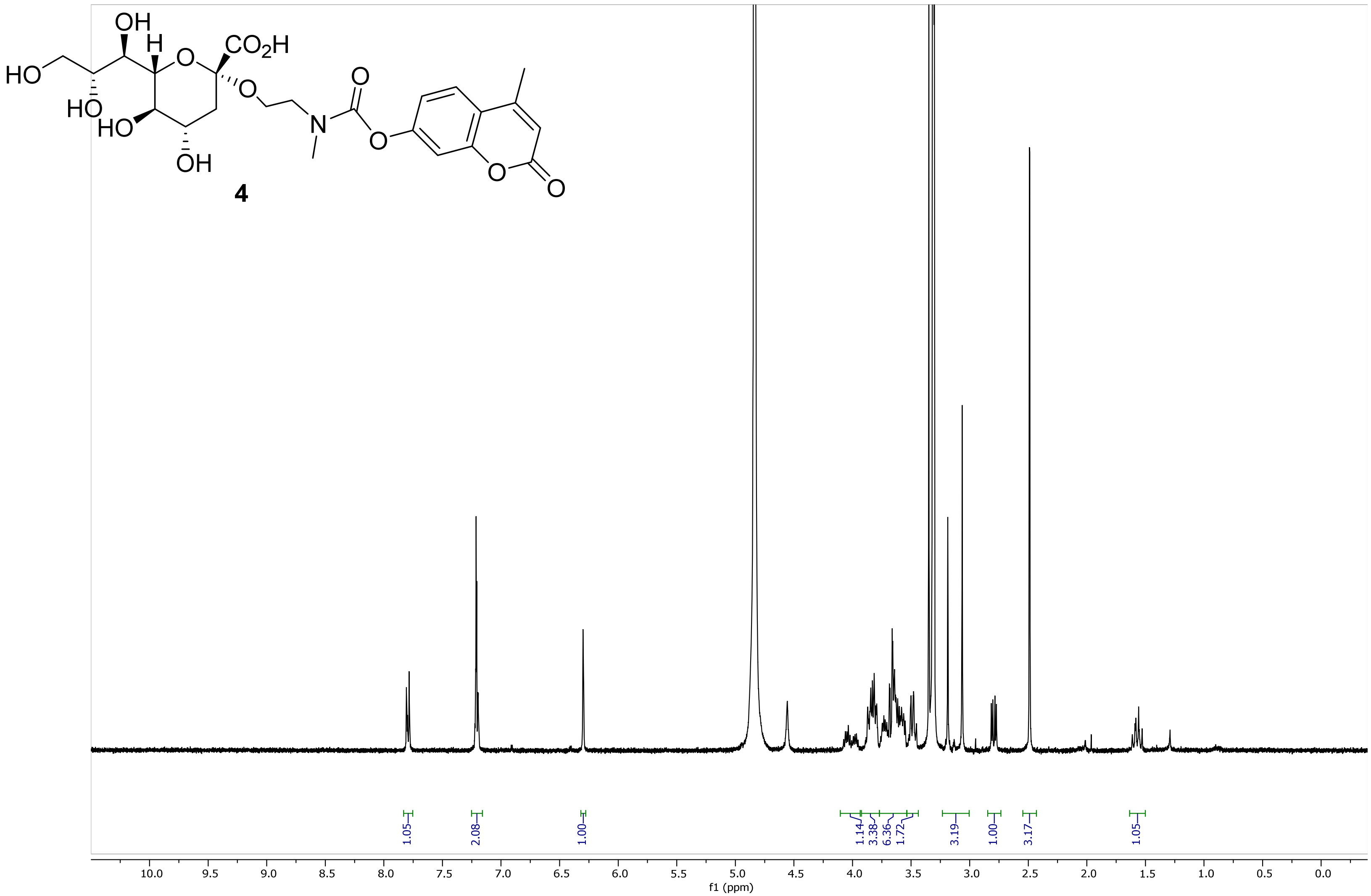

Figure S21. ^{1}H NMR spectrum of protected Kdn glycoside **4** in CD_3OH (400 MHz).

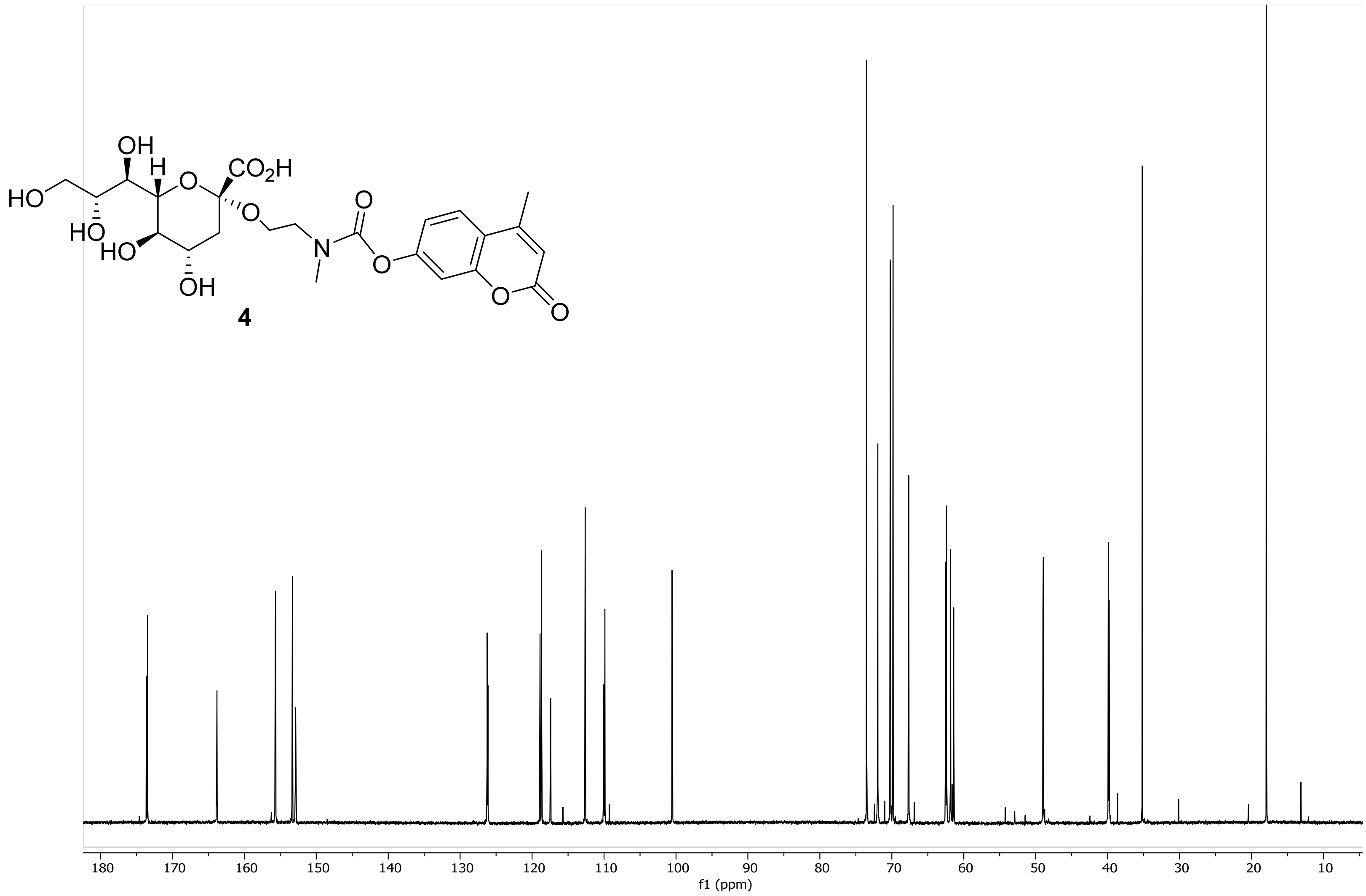

Figure S22. ^{13}C ^{1}HNMR spectrum of protected Kdn glycoside **4** in CD_3OH (101 MHz).
